# The effect of unintelligible speech noise on children’s verbal working memory performance

**DOI:** 10.3389/fpsyg.2025.1565112

**Published:** 2025-05-26

**Authors:** Gaia Spicciarelli, Flavia Gheller, Michael Celli, Barbara Arfé

**Affiliations:** ^1^Department of Developmental Psychology and Socialization, University of Padova, Padua, Italy; ^2^I-APPROVE Centre, University of Padova, Padua, Italy; ^3^Department of Computer, Control and Management Engineering, Sapienza University of Rome, Rome, Italy

**Keywords:** noise, children, working memory, attention, cognitive effort

## Abstract

Classroom noise, particularly speech noise from students, appears to disrupt verbal working memory processes essential for learning tasks such as reading and writing. Such negative interference has been alternatively explained as either the effect of phonological intrusion from speech noise into the phonological loop processes of working memory or the diversion of general attentional resources away from working memory processes. These effects have been studied primarily in relation to intelligible speech, while the impact of unintelligible speech, such as multitalker babble—a common source of noise in schools—has received less attention. The present study aimed to examine the effects of unintelligible multitalker babble noise on the performance of 38 students aged 8 to 10 years in low (Digit Span) and high demanding (Reading Span) verbal working memory tasks. We examined whether the effects of multitalker babble noise differed based on children’s age and their visual attentional resources. Verbal working memory and visual attentional skills were assessed in quiet and multitalker babble noise across three age groups: 8, 9, and 10 years. Our findings revealed that multitalker babble noise disrupts significantly verbal working memory performance in complex verbal working memory tasks, like the Reading Span task, but has minimal impact on low demanding tasks, like Digit Span Forward and Backward. Although children’s visual attentional skills significantly contributed to their verbal working memory performance, they alone cannot explain the negative effect of multitalker babble noise. Interestingly, although children’s behavioral performance did not significantly differ across acoustic conditions for most tasks, they reported higher cognitive effort in noisy environments. This highlights a discrepancy between perceived effort and actual performance, emphasizing the importance of integrating multiple measures to assess the impact of noise on cognitive processes.

## 1 Introduction

Exposure to noise is a well-known health risk factor for children, which is linked to a range of auditory and non-auditory health effects, including annoyance and impaired cognitive performance (Basner et al., 2014). Performing cognitively and linguistically complex academic tasks such as reading and writing can be challenging in noisy environments, especially with speech noise, as speech is an intrinsically relevant stimulus for both children and adults. Unfortunately, classrooms are often noisy learning settings, frequently characterized by babble (i.e., speech) noise generated by the children themselves.

Classroom babble, combined with noise from classroom activities such as hallway sounds or the scraping of chairs and tables, is considered one of the most annoying sources of noise for both teachers and students (Boman and Enmarker, 2004; Massonnié et al., 2022). Although the World Health Organization recommends a background noise limit of 35 dB(A) in classrooms and 55 dB(A) in recreation rooms (Berglund et al., 1999), the average sound level in classrooms often exceeds this threshold, reaching around 70 dB(A) (Lundquist et al., 2000; Shield and Dockrell, 2004; Sjödin et al., 2012). Noise levels, however, vary throughout the day. Quiet moments, such as during testing or assessment activities, alternate with peaks of noise during group work, where sound levels can even reach 130 dB(A)—surpassing the pain threshold (Sjödin et al., 2012).

Numerous studies have demonstrated that prolonged exposure to high levels of classroom noise can negatively impact academic performance of primary school children (aged 7 to 11 years) and adolescents (aged 11 to 16 years), affecting both non-verbal (e.g., memory and attention) and verbal tasks, with reading and spelling being particularly vulnerable to noise (Shield and Dockrell, 2008; Connolly et al., 2019). Furthermore, the detrimental effects of classroom noise extend beyond academic performance, affecting the psychophysical well-being and overall health of both children and teachers. Significant associations have been indeed observed between poor acoustical conditions and higher stress symptoms in both children aged 13 to 14 years (Boman and Enmarker, 2004) and teachers (Kristiansen et al., 2011; Grebennikov and Wiggins, 2006).

### 1.1 The impact of speech noise on children’s cognitive performance

Although research has shown that both environmental non-speech noise (such as traffic and heating, ventilation, and air conditioning systems) and speech noise can equally disrupt complex academic tasks such as reading, writing, and math (for a review, see Gheller et al., 2023), speech noise represents a primary source of interference in classrooms (Dockrell and Shield, 2006; Howard et al., 2010). Speech noise can consist of intelligible spoken words, such as in conversations between two speakers, or unintelligible babble noise, as in overlapping conversations, where the overlapping speech acts as a masker, generating an unintelligible verbal background. Its impact on children’s academic performance can be explained by the so-called Irrelevant Speech Effect (ISE) (Elliott, 2002). The ISE refers to the cognitive interference caused by background speech on individuals’ verbal working memory (WM), which negatively impacts their ability to process, maintain and recall information. Irrelevant speech is hypothesized to disrupt the verbal rehearsal mechanisms of the phonological loop, a key component of verbal working memory, reducing the capacity to temporarily maintain verbal information in tasks such as recalling word lists, reading, or writing (Elliott, 2002).

The cognitive level at which the ISE occurs remains uncertain. According to Klatte et al. (2010), the interference is at a linguistic level. The ISE arises because the phonological characteristics of irrelevant speech automatically engage the brain’s language processing centers including the phonological loop, thereby directly interfering with the rehearsal of linguistic information in verbal working memory. This interference, attributed both to the phonological properties of speech and its potential semantic content, leads to decreased recall performance and increased cognitive load (Klatte et al., 2013).

An alternative hypothesis is that the cognitive effect occurs at attentional level. According to this hypothesis, irrelevant speech diverts attentional resources needed for the working memory task, reducing the capacity to control working memory processes. Notably, this phenomenon would occur regardless of whether the interference is linguistic or non-linguistic (i.e., non-speech noises such as music or traffic). For this reason, this phenomenon was originally named “Irrelevant *Sound* Effect” (Jones et al., 1990).

The interference of speech noise has been studied in relation to simple verbal short-term memory tasks, such as digit span tasks (Elliott et al., 2016), and, more recently, in complex auditory-verbal memory tasks such as listening span tests, where speech noise may both interfere with the auditory items perception and their maintenance in working memory (Vettori et al., 2022). In contrast, less is known about the mechanisms underlying the irrelevant speech noise effect during complex verbal working memory tasks that do not involve speech perception, such as those required for reading (Gheller et al., 2023). Additionally, studies comparing the ISE across verbal working memory tasks with varying cognitive demands or complexity, such as digit span and reading span tasks, are lacking. To address these research gaps, this study aimed to compare elementary school children’s performance on digit span and reading span tasks, which require encoding and maintaining items visually presented in print. Given that many classroom activities impacted by noise involve reading or writing rather than listening (Shield and Dockrell, 2008), exploring the effects of speech noise on these tasks is crucial for assessing its impact on typical classroom activities.

#### 1.1.1 Developmental vulnerability and individual differences in response to classroom noise

The ability to ignore irrelevant stimuli to selectively attend relevant ones, along with the efficiency of memory processes, involves complex cognitive control mechanisms that develop with age (Gathercole et al., 2004; Scerif et al., 2012). The developmental immaturity of verbal memory and attentional processes may thus make young children more vulnerable to the ISE (Elliott et al., 2016; Jianxin and Peng, 2018). This can be particularly relevant for children still developing reading and spelling skills, which can be further hindered by poor classroom acoustics and speech-noise interference.

Factors such as individual sensitivity to noise and attentional abilities significantly influence how noise affects children’s cognitive performance. For instance, a recent study by Massonnié et al. (2022) found that primary school children aged 8 to 11 years with lower task-switching abilities reported increased interference and annoyance from noise. Additionally, children with a tendency toward mind-wandering (Kam, 2017) were more distracted by noise, though not necessarily more annoyed by it. These findings, consistent with earlier research (Boman and Enmarker, 2004; Stallen, 1999), indicate that annoyance and distraction from noise during learning activities are two related, yet distinct, constructs. Specifically, some children may be distracted by noise without feeling annoyed, or being fully aware of the interference, while others may report high annoyance without a corresponding decline in performance in the noise condition. On the one hand, these results suggest that children’s subjective perception of noise does not directly correlate with its actual impact on their cognitive performance (Massonnié et al., 2022; Hygge, 2003). On the other hand, they highlight the need to consider both self-reported judgments and behavioral data when studying the cognitive effects of noise, particularly in the youngest populations.

### 1.2 The effects of unintelligible speech noise on verbal working memory

Although intelligible speech is often considered more disruptive to cognitive performance than unintelligible speech, past research has shown that the ISE can occur regardless of whether the speech-noise is intelligible or not (Jones and Macken, 1993; Colle and Welsh, 1976). Intelligible speech noise can be more distracting because it contains semantic and phonological content that naturally engage not only attention, but also active speech encoding processes, leading to cognitive interference as the brain processes this irrelevant information alongside task-relevant one (Dockrell and Shield, 2006; Klatte et al., 2010). On the other hand, although unintelligible speech lacks both semantic content, and recognizable phonological components, its speech-like structure could still engage attention and the auditory processing pathways, competing with the processing resources needed for the task at hand (Jones and Macken, 1993; Hughes and Jones, 2001). However, the interference generated by unintelligible speech noise on verbal working memory remains poorly understood (Gheller et al., 2023).

Verbal working memory (VWM) is a memory system that temporarily holds verbal information, such as words, syllables or numbers, while also processing it to perform higher-order cognitive tasks like reading, reasoning, and problem-solving. Differently from visuospatial memory, for which visuo-spatial patterns rather than sequential order are crucial elements in performance, serial order of presentation is crucial in verbal memory (Donolato et al., 2017).

The key component of VWM is the phonological loop, which consists of two parts: the phonological store, a short-term storage which retains sequences of speech sounds in their auditory form for a few seconds, and an articulatory rehearsal process (the articulatory loop), which prevent the rapid decay of verbal memory traces by actively refreshing them through subvocal articulation (i.e., silently repeating sounds or words) (Baddeley, 2003).

The phonological loop operates under the supervision of the central executive, an attentional control system responsible for coordinating and allocating attentional resources across parallel processes and tasks. This supervision enables the execution of complex verbal cognitive tasks, such as language processing, reading comprehension, and verbal reasoning.

Evidence indicates that the phonological loop processes not only speech sounds but also non-speech sounds. Studies have shown that vocal music (Salamé and Baddeley, 1989), auditory tones (Jones and Macken, 1993) and instrumental music (Schweppe and Knigge, 2020), regardless of whether the music is liked or disliked (Perham and Vizard, 2011; Perham and Sykora, 2012; Bell et al., 2024) disrupt serial recall. These interference effects strongly suggest that the phonological loop automatically processes both auditory speech and non-speech information external to the verbal memory task during verbal memory performance (Jones and Macken, 1993).

However, less is known whether this type of noise affects cognitive processes that do not require listening or auditory processing, such as reading tasks. A recent study by Guerra and colleagues (Guerra et al., 2021) investigated the impact of intelligible and unintelligible speech noise on reading performance in third and fourth graders. Their findings revealed that reading speed was not significantly affected by noise intelligibility; however, intelligible speech noise impaired reading comprehension, leading to poorer performance compared to unintelligible speech noise.

This finding aligns with previous studies on adults (Martin et al., 1988; Vasilev et al., 2019), suggesting that the semantic content of speech triggers automatic processing of irrelevant semantic input, with this semantic interference disrupting reading performance.

A key methodological issue in studying the effects of speech noise on verbal working memory is selecting an appropriate task for assessing verbal working memory performance. Digit Span tasks are the most commonly used verbal working memory tasks, as they provide simple measures of verbal working memory capacity. The standard Digit Span task consists of two subtests: Forward and Backward Digit Span. The Forward Digit Span subtest, which involves recalling sequences of spoken digits of increasing length, is used to assess serial recall and the efficiency of the verbal rehearsal processes within the phonological loop (Acheson and MacDonald, 2009). In contrast, the Backward Digit Span, which requires recalling digit sequences in reverse order, is considered a measure of the cognitive, executive, control mechanisms of working memory (Giofrè et al., 2016; Alloway et al., 2004). The different cognitive underpinnings of the forward and backward verbal memory tasks seem supported by studies showing that the two tasks also have different neural correlates (Donolato et al., 2017). For instance, performance on the Backward Digit Span task is associated with the activation dorsolateral and frontal regions that are typically activated in tasks requiring cognitive control.

In addition to the traditional spoken/auditory version of the task, computerized versions have been developed involving the repetition of sequences of single digits visually presented one at a time on a screen (Olsthoorn et al., 2014; Tractenberg and Freas, 2007). Despite differences in mode of presentation, the two versions convey similar patterns of results (Olsthoorn et al., 2014) and appear to measure the same constructs (Tractenberg and Freas, 2007). In particular, the superior performance in forward recall compared to backward recall, which is typical of verbal memory, is observed in both auditory and visual digit span tasks (Olsthoorn et al., 2014; Tractenberg and Freas, 2007).

Another widely used task for assessing verbal working memory capacity, particularly in relation to complex verbal activities like reading comprehension, is the Reading Span task, introduced by Daneman and Carpenter (1980). This task involves processing (comprehending) sets of sentences while simultaneously temporarily storing the final word of each sentence for subsequent recall. Since the Reading Span task involves both temporary storage and processing of complex verbal information (sentences), it demands greater executive resources than simpler verbal WM tasks such as the Digit Span (Arfé et al., 2015). Compared to the Digit Span, the Reading Span task is thus considered a better indicator of the executive control component of verbal WM (Arfé, 2015; Arfé and Fastelli, 2020). Comparing noise interference on individuals’ performance on these verbal working memory tasks (Forward Digit Span, Backward Digit Span and Reading Span) could help identify which verbal WM component—verbal rehearsal and phonological loop or central executive—is most affected by the interference of unintelligible speech noise.

### 1.3 Aims and hypotheses

The study aimed to examine the effects of unintelligible background speech noise on the verbal working memory performance of children aged 8 to 10 years. To investigate the nature of these effects, children’s verbal working memory was examined in quiet and noise conditions across three working memory tasks: Forward Digit Span, Backward Digit Span and Reading Span. The role played by sustained visual attention skills on children’s performance was also explored, to account for the contribution of domain-general sustained attentional resources to children’s verbal working memory performance in quiet and noise.

The study addressed three research questions:

1. Does unintelligible multi-talker babble noise affect 8-to-10-year-old children’s verbal working memory performance across verbal working memory tasks? Is its interference mainly at the level of the phonological loop or the executive control mechanisms of verbal WM?
2. Does the effect of unintelligible speech noise vary with children’s age and working memory skills?
3. Do sustained visual attention skills contribute to explain the effects of multi-talker babble noise on verbal working memory tasks?

The verbal working memory and visual attentional skills of 8- to 10-year-olds were assessed in both quiet and noisy conditions using a novel tablet application, “CoEN: Cognitive Effort in Noise” (Arfé et al., 2022; Gheller et al., 2024).

Children from grade 3 to 5 were recruited, as by grade 3 children’s reading abilities typically progress beyond the initial learning phase. Younger children in earlier grades were deemed less suitable for this study because consolidated reading skills were required to perform the reading span task. Although Italian is a shallow orthography, first and second graders are not yet fluent readers (Zoccolotti et al., 2009).

Participants were divided in three age groups—8, 9, and 10 years—to account for the maturation of their cognitive skills (Gathercole et al., 2004). Given that verbal working memory develops significantly with maturation and learning (Gathercole et al., 2004), our study aimed to examine whether the effects of unintelligible babble noise differed across the three age groups, reflecting the development of these essential skills.

As anticipated, previous research highlighted that both intelligible and unintelligible speech noises can interfere with performance on both working memory and academic tasks (Gheller et al., 2023). However, the specific cognitive locus (i.e., phonological loop or attentional mechanism) of the ISE on working memory remains unknown. The hypothesis of this study was that unintelligible multi-talker babbling, a phonologically and socially relevant stimulus for children, would negatively impact children’s verbal WM performance by detracting their attentional resources from the verbal WM task, and that the interference would be greater for cognitively demanding tasks, such as the Reading Span task, compared to less demanding tasks like the Digit Span task. In line with this hypothesis, we expected a significant (domain-general) contribution of visual attention to children’s performance on the most complex verbal WM tasks. Moreover, since the mastery of the executive control mechanisms of verbal WM increases with age, we anticipated a greater interference from multi-talker babbling in younger than in older children. In contrast, we did not have specific predictions regarding the interference of unintelligible multi-talker babbling on the phonological loop mechanisms of WM.

## 2 Materials and methods

### 2.1 Participants

The study initially involved 51 children (mean age = 9.04 years, SD = 0.824; 25 girls), aged 8 to 10 years, from three classes (third, fourth and fifth grades) of a public primary school in central Italy. Prior to data collection, written informed consent was obtained from the parents of all participants, allowing their children to take part in the study.

Following data collection, participants were screened based on predefined criteria to ensure comparability across the sample. Specifically, all participants had to be native Italian speakers or, for children from immigrant families, either born in Italy or enrolled in Italian schools for at least 3 years. Children with certified learning or developmental disabilities or any sensory impairments (i.e., visual or hearing impairments) were excluded. In addition, children receiving support from a special education teacher or children whose teachers reported having special educational needs—including children undergoing evaluation for suspected learning or developmental disorders—were also excluded from the final sample.

After applying these criteria, the sample consisted of 41 children (mean age = 9.02 years, SD = 0.851; 20 girls). A description of additional data exclusions based on data quality checks and task performance is provided in the Results section.

### 2.2 Procedure

All children individually performed four tasks assessing verbal working memory and attention twice at one-week interval, using a novel tablet application called “CoEN: Cognitive Effort in Noise” (Arfé et al., 2022; Gheller et al., 2024). The App integrates five standardized neuropsychological tasks, including selective and sustained visual attention tasks and verbal WM tasks such as Digit Span and Reading Span (Wechsler, 2003; Arfé et al., 2015), which have been adapted from paper-and-pencil versions into a digital, game-like format. In this study children performed only four tasks of the App, which were always presented in a fixed order within each session. The order of presentation was as follows: the Digit Span Task (Forward and Backward conditions), adapted from the WISC-IV battery (Wechsler, 2003); an Italian Reading Span test, adapted from the original Daneman and Carpenter’s task (Daneman and Carpenter, 1980; Arfé et al., 2015); a visual search task adapted from the NEPSY-II battery (Korkman et al., 2011); a visual attention task adapted from the cancellation subtest of the WISC-IV battery (Wechsler, 2003).

Children completed all tasks twice: once in quiet and once while exposed to unintelligible multi-talker babble noise. The order of acoustic conditions was counterbalanced across participants. Both testing sessions took place at school in a quiet room at one-week interval in order to minimize learning effects.

The tasks were performed by each child individually in a quiet room during school hours. The researcher introduced the App CoEN and the tasks to the child. Each task within the tablet application was also introduced by on-screen instructions and a brief practice block, which the child could repeat as many times as needed until ready to proceed. The researcher monitored the entire session to ensure the child was comfortable and to assist with any technical issues related to the tablet application, but no assistance or feedback was provided during task performance. Each session lasted approximately 30 to 45 min, with brief breaks provided between tasks if requested by the child.

In the quiet condition, all tests were performed in a room distant from noise sources. Children wore over-ear headphones (AKG K240 model) to further isolate them from potential sources of environmental noise (e.g., air conditioning or ventilation, or corridor noise). In the noise condition, children performed the tests in the same room, wearing the same over-ear headphones (AKG K240 model), through which unintelligible multi-talker babble was played at 65 dB(A), representing the average conversation level (Mama et al., 2018). The audio file used was the same one commonly employed in clinical settings for vocal audiometry tests (Cutugno et al., 2000).

To ensure that the actual output signal intensity matched the level specified on the tablet, the device was calibrated using an artificial ear equipped with a headphone mechanical coupler in combination with an oscilloscope. Although the same headphone model mentioned earlier was always used in the study, the calibration was conducted for various headphone classes to provide guidance on how to adjust the tablet according to the impedance and dynamic range characteristics of the headphones.

At the end of each session, children completed a paper-and-pencil cognitive effort self-report questionnaire (Bess et al., 2014) to assess their perceived fatigue following the tasks. The questionnaire consisted of six statements (e.g., “I felt tired”) accompanied by illustrative drawings to aid comprehension, and the child was asked to rate each statement on a five point Likert Scale (from “not at all” to “a lot”). This self-report measure was included to explore whether children experienced greater cognitive effort in the noise condition compared to the quiet condition.

### 2.3 Measures

Verbal working memory measures were the dependent variables in this study. Selective and sustained visual attention measures served as control variables to determine whether any potential negative effects of babble noise could be related to children’s domain-general attentional resources.

#### 2.3.1 Digit span test

The Digit Span task evaluates children’s verbal working memory capacity. In the original test (Wechsler, 2003), spoken digits are presented to the participant, who is then asked to recall them in the same order as presented (forward subtest) or in the reverse one (backward subtest). To assess cognitive processes independently of listening-in-noise abilities, in the CoEN App we adapted the task by visually presenting digits one at a time on the screen at a rate of one per second. This adaptation ensured that the assessment of children’s working memory was not influenced by their ability to hear amidst noise, enabling a more accurate evaluation of their cognitive performance. Children were instructed to type the sequences they observed using the App touchscreen keyboard, either in the same order for the forward subtest or in reverse order for the backward subtest. As in the original Digit Span task (Wechsler, 2003), two sequences of the same length were administered at each span level. The task followed the standard discontinuation rule, according to which the task ends when the child fails to accurately recall two consecutive sequences of the same length.

*Scoring*. We employed the scoring procedure suggested by Conway et al. (2005), which has been shown to be a more reliable measure of working memory abilities in Digit Span tasks compared to the standard span score, particularly for the Digit Span Backward (Weitzner et al., 2022; Wells et al., 2018). Unlike the traditional all-or-nothing scoring method, where participants are awarded zero points for any sequence with errors –whether due to omitting a single digit or recalling the entire sequence incorrectly (Wechsler, 2003)—the alternative method assigns a partial credit for any correct digit recalled. Specifically, each digit recalled in the correct serial position is awarded one point (Conway et al., 2005; Wells et al., 2018; Weitzner et al., 2022). This approach allows participants to receive partial credit for trials even if they do not recall all digits correctly.

In the Digit Span Forward task, sequences ranged from 2 to 9 digits, with two trials at each span length, leading to a maximum possible score of 88 points. In the Digit Span Backward task, sequences ranged from 2 to 8 digits, also with two trials per span length, resulting in a maximum possible score of 74 points.

While reflecting both the storage and manipulation aspects of verbal working memory, this scoring ensures a more precise and sensitive measure of digit span capacity compared to the all-or-nothing scoring system.

#### 2.3.2 Reading span test

The Reading Span task (Arfé et al., 2015) requires participants to simultaneously comprehend short sentences to answer true/false questions and store the final word of each sentence for later recall. The sentences were visually presented one at a time on the screen, organized into series (or blocks) that progressively increased in length from 2 to 5 sentences, for a total of 28 sentences. After completing each series, the child was required to recall all the final words from the sentences in the correct order (Arfé et al., 2015). Children were instructed to write down all the words they remembered, even if they were unsure about the sequence. This instruction was specifically designed to ensure that uncertainty about the word order would not deter children from reporting words they recalled, thereby providing a more accurate measure of their memory span. A block was considered successfully completed if the child correctly recalled all the final words from the sentences in the block. The task ended when the participant failed two consecutive blocks of the same length.

*Scoring*. The number of correctly recalled sentence sequences (i.e., trials) was considered as the score. Two trials for each span length (2, 3, 4 and 5 sentences) were presented, resulting in a total of 8 trials. Participants were awarded one point for each correctly recalled final word sequence, with a maximum possible score of 8. A sequence was considered correct if all the words were recalled, regardless of their exact order of presentation. All correct responses were accepted, even if they contained minor typos, such as omission of a geminate (‘orechie’ for ‘orecchie’—*ears*) or a single-letter substitution (‘puaderni’/‘cuaderni’ for ‘quaderni’—*notebooks*). This procedure ensured that a single spelling mistake did not negatively affect scoring. Performance was scored only for children who achieved at least 75% accuracy on the comprehension task. Given the simplicity of the sentences, we interpreted a performance below this threshold as unreliable, likely due careless reading or insufficient attention (Arfé et al., 2015).

#### 2.3.3 Visual search task (faces)

Visual search tests are effective tools for assessing selective sustained attention, as they require the ability to maintain attentional control to selectively process stimulus features—a skill that develops early in childhood (Welsh et al., 1991). Adapted from the NEPSY-II battery (Korkman et al., 2011), this visual search task required children to quickly and accurately scan matrices of face drawings, identifying target faces with specific features, distinguishing them from distractor faces that varied in characteristics such as hairstyle, or gaze direction. The task lasted 3 min, challenging children to efficiently discern the relevant features amidst similar stimuli. The scoring procedure is reported below.

#### 2.3.4 Cancellation task (animals)

The cancellation task, adapted from the WISC-IV battery (Wechsler, 2003), was designed to assess rapid visual scanning and processing skills in children. Similar to the visual search task, it required children to identify specific target items—animals—amidst a variety of distracting stimuli, including vegetables, fruits, tools, and clothing. However, this task was shorter in duration, lasting only 45 s, thereby placing greater emphasis on speed and immediate recognition. While the original WISC-IV task involved two conditions, random and structured, we chose to adapt only the structured condition (i.e., where targets and non-targets stimuli are arranged in a structured and fixed pattern). This decision was informed by evidence suggesting that the structured condition is particularly sensitive for assessing attention in children, including those with attentional problems (Zhu and Chen, 2013).

#### 2.3.5 Scoring of the two attention tasks

Signal Detection Theory (SDT) was used as a framework for determining the scoring criteria of the Visual Search and Cancellation tasks. The theory examines how individuals make detection decisions to discriminate target stimuli from non-target stimuli under conditions of uncertainty (Stanislaw and Todorov, 1999). According to the SDT, the detection of a target depends on both the intensity of the stimulus and the observer’s sensory threshold and decision-making strategies. Two central constructs of SDT are sensitivity and decision criterion. Sensitivity (*d’*) measures the observer’s ability to distinguish target stimuli from non-targets, while the decision criterion (**C**) represents the threshold at which a signal is identified as present.

Within this framework, children’s responses were categorized as follows:

1. *Hit*: a correct response where the child accurately identifies a target (e.g., selecting an animal in the cancellation task or a correct face in the visual search task);
2. *False Alarm*: an incorrect response that occurs when the child incorrectly identifies a non-target as a target (e.g., selecting a distractor face in the visual search task or selecting an object instead of an animal in the cancellation task);
3. *Miss*: an omitted response, when the child simply fails to detect the target (e.g., the child does not identify and select the target face or the animal when present in the cancellation task);
4. *Correct Rejection*: a correct response, recorded when the child appropriately avoids selecting non-target stimuli (e.g., correctly ignoring a face distractor or an object when only animals should be selected). This is calculated separately from the *hits*.

From these response categories, sensitivity (*d’*) and criterion (**C**) measures were derived. Sensitivity (*d’*) reflects the ability to distinguish targets from non-targets. It is calculated as the difference between the z-transformed hit rate and the z-transformed false alarm rate (*Hit rate*—*False Alarm rate*). The *Hit rate* is the number of correctly identified targets divided by the total number of targets presented, representing the proportion of correctly identified targets (*Hit rate = Number of Hits / Total Number of Targets*). The False Alarm Rate is calculated by dividing the number of incorrect responses by the total number of non-target trials, reflecting the proportion of non-targets that are incorrectly identified as targets (*False alarm rate = Number of False Alarms / Total Number of Non-Targets*). Higher *d’* values indicate better discrimination skills (i.e., the child is better at distinguishing targets—such as animals or correct faces—from the non-targets—such as objects or distractor faces).

The Criterion (**C**) measure, by contrast, reflects the participants’ decision bias, which essentially corresponds to the threshold level at which they decide whether a stimulus is a target or a non-target. **C** is calculated as the negative average of the z-scores for the *hit rate* and the *false alarm rate*. Negative values of **C** (**C** < 0) suggest that the participant is more likely to say “*yes*” and identify stimuli as targets, while positive values (**C** > 0) indicate a more cautious approach, with a tendency to say “*no*” and identify fewer stimuli as targets. Finally, **C** values around zero indicate no significant bias toward either “*yes*” or “*no*” responses, reflecting a balanced approach to decision-making, where the participant is equally cautious in identifying a stimulus as target or rejecting non-targets. Such approach reflects unbiased judgment under conditions of uncertainty.

Sensitivity (*d’*) and criterion (**C**) measures were computed separately for each of the two attentional tasks and conditions. Subsequently, mean *d’* and **C** scores were calculated by averaging the scores for the two attention tasks in quiet and in noise, resulting in four attentional measures: *d’* mean Quiet and *d’* mean Noise, **C** mean Quiet and **C** mean Noise.

Applying SDT allows for an in-depth analysis of children’s visual attention capabilities by examining not just accuracy, but also their decision-making tendencies—whether they tend to over-respond or are overly cautious.

#### 2.3.6 Familiarity with digital technology questionnaire and cognitive effort self-report

Before starting the experiment, children completed a short six-item multiple-choice questionnaire designed to assess their familiarity with various digital technologies, including personal computers, smartphones, tablets, and gaming consoles. Nearly all participants (98.68%) reported regular engagement with touchscreen devices, at least once per week.

At the end of each session, children filled out a *Cognitive effort* questionnaire (Bess et al., 2014), adapted to Italian, to assess their perceived cognitive fatigue after the tasks. The original questionnaire included five statements, each rated on a five-point Likert scale ranging from 1 (not at all) to 5 (very much), accompanied by illustrative drawings to facilitate comprehension. The statements assessed different aspects of perceived fatigue, including physical sensations (e.g., “My head hurts,” “I feel tired”), concentration difficulties (e.g., “It is hard for me to pay attention”) and cognitive load (e.g., “I have trouble thinking,” “It is easy for me to do these things”). We added a sixth statement “I was distracted by noise,” in order to investigate children’s subjective perception of noise interference during the sessions.

The mean score across these items indicated the participant’s overall perceived cognitive fatigue, with higher values reflecting greater fatigue. We anticipated higher scores after the noisy condition.

### 2.4 Statistical approach

Two sets of analyses were conducted to answer the three research questions of the study:

1. *Does unintelligible multi-talker babble noise affect 8-to-10-year-old children’s verbal working memory performance and is its interference mainly at the level of the phonological loop or the executive control component of verbal WM?*
2. *Does the effect of unintelligible speech noise* var*y with children’s age?*
3. *Do sustained visual attention skills contribute to explain the effects of multi-talker babble noise on verbal working memory tasks?*

The first two research questions aimed to explore whether unintelligible multi-talker speech noise affected children’s verbal working memory performance, the main locus of its interference (the phonological loop or executive control component of verbal WM), and whether the interference effect varied with children’s age. The third research question aimed to further explore whether the attentional interference of unintelligible speech was related to domain-general attentional resources or not.

We first run preliminary analyses of variance (mixed ANOVA) to test whether children actually perceived greater cognitive fatigue (i.e., reported higher cognitive load) in the noise condition. The mean cognitive effort score from the self-report fatigue questionnaire was used as the dependent measure, while condition (quiet/noise) and age group (8, 9, and 10 years) were used as the independent factors.

To address research questions 1 and 2, a linear mixed-effects model (LMM) was employed to test the effects of acoustic condition (quiet or babble noise), age (8, 9, and 10 years) and WM task type (Digit Span Forward, Digit Span Backward, Reading Span) on children’s verbal WM performance. To compute a single verbal WM measure, the raw scores from each of the three verbal working memory tasks were transformed into z-scores using the overall sample’s mean and standard deviation. Participants were included as a random factor to account for individual variability.

To address research question 3, visual attention scores were included as covariates in task-specific mixed models to explore whether sustained visual attention skills contributed to explain the effects of multi-talker babble noise on children’s verbal WM performance. The contribution of visual attention was considered only for WM tasks for which significant noise effects were observed. Linear mixed models were computed using GAMLj3 for Jamovi (v. 2.6.2) (Gallucci, 2019; The jamovi project, 2024).

As one two of our factors (age and WM task type) had three levels (8, 9 and 10 years; DS Forward, DS Backward, Reading Span), the variables age, acoustic condition and WM task type were defined as categorical and coded using *effect* coding (Brehm and Alday, 2022). *Effect* (or *sum* or *contrast*) coding is a technique that compares each level of a categorical variable to the overall average (the grand mean). This approach is particularly useful when one variable has more than two levels and is often considered a more appropriate choice compared to the default *dummy* coding. In *dummy* coding, each level is compared to a designed reference level, with coefficient estimating the difference in the dependent variable between the reference category and the other categories. However, as the comparison is always made between the other levels and the chosen reference level, changing the reference category would also change the estimated effects. *Effect* coding, on the other hand, estimates how much each level deviates from the overall trend (grand mean), rather than from a particular reference level. Since the grand mean remains constant regardless of the chosen reference level, the results of effect coding are considered more stable and independent of the choice of reference. For this study, the factor coding option *deviation* in Jamovi was used to effect-code the categorical variables (Brysbaert and Debeer, 2025).

Descriptive statistics for working memory, visual attention, and cognitive effort measures across the three age groups are reported in Table 1. In Tables 2–4 the fixed effects parameter estimates are reported including 95% confidence intervals and statistical significance for each parameter. The goodness of fit of the mixed-effects models is assessed using marginal and conditional R^2^, which represent the variance explained by fixed effects alone and by both fixed and random effects, respectively (Nakagawa and Schielzeth, 2013; Brysbaert and Debeer, 2025). Additionally, we report the intra-class correlation coefficient (ICC) to reflect the proportion of random variance attributable to participants, along with the variance explained by participant intercepts (τ_00_) and the residual variance (σ^2^).

**Table 1:** Descriptive statistics for verbal working memory, visual attention and cognitive effort (self-report) measures in quiet and noise.

Task	Age group	*N*	Mean	SD	Min	Max	Skewness	Kurtosis
Quiet—DS Forward (Partial score)	8 years	12	25.33	7.54	17	41	−0.239	0.785
	9 years	12	27.67	9.24	6	43		
	10 years	14	28.07	5.62	17	37		
Noise—DS Forward (Partial score)	8 years	12	26.67	6.68	18	37	−0.416	1.139
	9 years	12	24.00	10.63	3	35		
	10 years	13	30.15	8.72	18	45		
Quiet—DS Backward (Partial score)	8 years	12	12.50	7.21	0	22	−0.365	0.808
	9 years	12	22.42	5.66	17	34		
	10 years	14	22.86	6.25	16	35		
Noise—DS Backward (Partial score)	8 years	12	18.33	7.80	3	35	1.553	4.444
	9 years	12	24.17	9.71	11	47		
	10 years	14	23.57	9.19	17	54		
Quiet—Reading Span score	8 years	12	2.42	1.68	0	6	−0.087	0.034
	9 years	12	3.50	0.91	2	5		
	10 years	14	3.29	1.44	1	6		
Noise—Reading Span score	8 years	12	1.58	1.56	0	5	0.576	0.095
	9 years	12	3.08	1.73	1	7		
	10 years	14	2.71	1.44	0	5		
Quiet—*d’* mean	8 years	12	2.09	0.46	1.01	2.72	−0.697	1.330
	9 years	12	2.40	0.47	1.66	3.20		
	10 years	14	2.37	0.57	0.82	3.11		
Noise—*d’* mean	8 years	12	2.04	0.44	0.88	2.58	−0.500	0.591
	9 years	12	2.50	0.37	1.79	2.96		
	10 years	14	2.34	0.53	1.52	3.20		
Quiet—Criterion mean	8 years	12	1.23	0.38	0.50	1.88	0.296	−0.085
	9 years	12	1.14	0.29	0.75	1.72		
	10 years	14	1.05	0.28	0.54	1.76		
Noise—Criterion mean	8 years	12	1.17	0.28	0.68	1.58	−0.244	−0.633
	9 years	12	0.98	0.29	0.60	1.44		
	10 years	14	1.03	0.25	0.42	1.39		
Quiet—Self Report mean	8 years	12	1.65	0.68	1.00	3.33	0.814	1.313
	9 years	12	2.06	0.61	1.17	3.50		
	10 years	14	1.80	0.43	1.00	2.50		
Noise—Self Report mean	8 years	12	1.99	0.40	1.00	2.67	0.328	0.359
	9 years	12	2.29	0.69	1.17	3.67		
	10 years	14	1.95	0.57	1.00	3.00		

**Table 2:** Parameter estimates from the Linear Mixed-Effects model: effects of acoustic condition, age and WM task type on working memory performance (*N* = 38).

Fixed effects	Estimates	95% CI Lower	95% CI Upper	*p*-value
(Intercept)	−0.011	−0.197	0.174	0.906
Age Group (9 years)	0.169	−0.096	0.435	0.217
Age Group (10 years)	0.237	−0.019	0.493	0.076
Acoustic Condition (Noise)	−0.013	−0.122	0.096	0.817
WM Task (Backward Digit Span)	−0.005	−0.159	0.150	0.953
WM Task (Reading Span)	0.003	−0.151	0.157	0.967
Age Group (9 years) * Acoustic Condition (Noise)	−0.072	−0.229	0.084	0.361
Age Group (10 years) * Acoustic Condition (Noise)	0.007	−0.143	0.158	0.924
Age Group (9 years) * WM Task (DS Backward)	0.141	−0.080	0.362	0.209
Age Group (10 years) * WM Task (DS Backward)	0.064	−0.149	0.277	0.553
Age Group (9 years) * WM Task (Reading Span)	0.168	−0.052	0.389	0.135
Age Group (10 years) * WM Task (Reading Span)	−0.086	−0.299	0.127	0.426
Acoustic Condition (Noise) * WM Task (DS Backward)	0.175	0.021	0.329	0.027
Acoustic Condition (Noise) * WM Task (Reading Span)	−0.181	−0.336	−0.027	0.022
Age Group (9 years) * Acoustic Condition (Noise) * WM Task (DS Backward)	0.013	−0.208	0.234	0.908
Age Group (10 years) * Acoustic Condition (Noise) * WM Task (DS Backward)	−0.127	−0.340	0.085	0.239
Age Group (9 years) * Acoustic Condition (Noise) * WM Task (Reading Span)	0.133	−0.087	0.354	0.235
Age Group (10 years) * Acoustic Condition (Noise) * WM Task (Reading Span)	0.004	−0.209	0.217	0.970
Random effects				
σ^2^ (Residual)	0.692			
τ_00_ (Participant Intercept)	0.219			
ICC (Intraclass Correlation)	0.240			
**Marginal R^2^/Conditional R^2^**	0.140/0.346			

Fixed effects estimates represent deviations from the grand mean. The baseline levels—8 years for Age Group, Digit Span Forward for WM Task, and quiet for Acoustic Condition—are omitted; estimates for the other levels indicate differences relative to these baselines. Positive estimates indicate higher performance, while negative estimates indicate lower performance.

**Table 3:** Linear Mixed-Effects model for the Reading Span task with *d’* mean as a covariate (*N* = 38).

Fixed effects	Estimates	95% CI	*p*-value
(Intercept)	2.768	[2.372, 3.165]	<0.001
Age Group (9 years)	0.400	[−0.180, 0.981]	0.177
Age Group (10 years)	0.173	[−0.376, 0.721]	0.534
Acoustic Condition (Noise)	−0.308	[−0.556, −0.059]	0.019
*d’* mean	0.914	[0.200, 1.628]	0.013
Age Group (9 years) * Acoustic Condition (Noise)	0.121	[−0.249, 0.492]	0.517
Age Group (10 years years) * Acoustic Condition (Noise)	0.061	[−0.284, 0.405]	0.727
Acoustic Condition (Noise) * *d’* mean	−0.424	[−1.016, 0.169]	0.161
Random effects			
σ^2^ (Residual)	1.169		
τ_00_ (Participant Intercept)	0.906		
ICC (Intraclass Correlation)	0.437		
**Marginal R^2^/Conditional R^2^**	0.234/0.569		

Fixed effects estimates represent deviations from the grand mean. The baseline levels—8 years for Age Group and quiet for Acoustic Condition—are omitted; estimates for the other levels indicate differences relative to these baselines. Positive estimates indicate higher performance, while negative estimates indicate lower performance.

**Table 4:** Linear Mixed-Effects model for the Reading Span task with C mean as a covariate (*N* = 38).

Fixed effects	Estimates	95% CI	*p*-value
(Intercept)	2.761	[2.377, 3.144]	<0.001
Age Group (9 years)	0.458	[−0.092, 1.008]	0.105
Age Group (10 years)	0.134	[−0.397, 0.666]	0.617
Acoustic Condition (Noise)	−0.743	[−1.274, −0.213]	0.008
C mean	−1.706	[−2.892, −0.519]	0.005
Age Group (9 years) * Acoustic Condition (Noise)	0.038	[−0.720, 0.796]	0.921
Age Group (10 years years) * Acoustic Condition (Noise)	0.109	[−0.623, 0.840]	0.768
Acoustic Condition (Noise) * C mean	−0.441	[−2.425, 1.542]	0.659
Random effects			
σ^2^ (Residual)	1.291		
τ_00_ (Participant Intercept)	0.734		
ICC (Intraclass Correlation)	0.363		
**Marginal R^2^/Conditional R^2^**	0.238/0.514		

Fixed effects estimates represent deviations from the grand mean. The baseline levels—8 years for Age Group and quiet for Acoustic Condition—are omitted; estimates for the other levels indicate differences relative to these baselines. Positive estimates indicate higher performance, while negative estimates indicate lower performance.

In the text, we report the main effects of acoustic condition, age, WM task type and their interaction based on the fixed-effects ANOVA outputs provided by the GAMLj3 package’s LMM analysis. This ANOVA serves as the omnibus test for evaluating the overall significance of the effects and interactions across all levels of the factors. Instead of reporting all default *post hoc* pairwise comparisons tested by Jamovi, in Supplementary material we report a-priori pairwise comparisons based on our specific hypotheses, which were computed using jamovi’s custom contrasts (via the Rj Editor), thereby reducing the risk of false positives (type I errors) (Granziol et al., 2025).

## 3 Results

### 3.1 Descriptive statistics and preliminary analyses

Of the 41 children meeting the inclusion criteria, one child was excluded from the analysis due to technical issues with the tablet application that resulted in his performance not being correctly recorded. Additionally, two children were excluded for achieving a true/false comprehension accuracy below 75% at the Reading Span task, a level suggesting inadequate attention or careless reading.

Consequently, the final sample consisted of 38 children (mean age = 9.05 years, SD = 0.837; 19 girls), divided into three age groups: 12 children aged 8 years (5 girls), 12 children aged 9 years (7 girls), and 14 children aged 10 years (7 girls). Table 1 presents the descriptive statistics for working memory, attention and cogntiive effort measures.

In line with prior research, all age groups showed comparatively a better performance on the quiet digit forward than on the quiet digit backward task (Donolato et al., 2017).

Mean *d’* values ranged from approximately 2 to 2.5 in both acoustic conditions for all age groups, indicating that children’s sensitivity remained relatively stable, even in presence of noise.

Regarding Criterion, mean values were positive (C > 0) in both acoustic conditions for all age groups, indicating that children generally adopted a cautious response approach. Values of skewness and kurtosis were acceptable for all measures, with the exception of Digit Span Backward, which displayed relatively high kurtosis in noise, indicating some extremely low scores. In quiet, the distribution was more balanced.

Self-reported cognitive effort scores in noise were higher than in quiet, with a large effect size: *F*(1, 35) = 7.029, *p* = 0.012, η_p_^2^ = 0.167, indicating greater perceived fatigue in the noise compared to the quiet condition. The interaction between age and acoustic condition was not significant, suggesting that the higher perceived effort in the noise condition was consistent across all three age groups.

### 3.2 Effects of noise condition on verbal WM performance

A linear mixed-effects model was conducted to assess the effects of acoustic condition, age and WM task type (Digit Span Forward and Backward, and Reading Span) on working memory performance.

Age (with three levels—8, 9, and 10 years), acoustic condition (with two levels—quiet and noise) and WM task type (with three levels—DS Forward, DS Backward, Reading Span) were included as independent factors (fixed effects), while z transformed WM score was the dependent measure.

The omnibus ANOVA revealed that the effect of acoustic condition was not significant, suggesting that children’s overall performance across all memory tasks was not significantly affected by noise. The main effect of WM task type was also not significant, indicating that performance did not differ significantly across the three WM tasks.

In contrast, the main effect of age group was significant, *F*(2,36) = 4.651, *p* = 0.016. Children’s Pairwise comparisons (Supplementary Table 1S) revealed that 8-year-old children performed significantly worse than both 9-year-olds (*p* = 0.020) and 10-year-olds (*p* = 0.008). No significant difference was observed between the 9-year-olds and the 10-year-olds.

The interaction between age and WM tasks type was significant, *F*(4, 174) = 2.508, *p* = 0.044, showing that performance across different working memory tasks varied depending on the children’s age. Pairwise comparisons (Supplementary Table 2S) revealed that 8-year-old children performed significantly worse in the Digit Span Backward task compared to 9-year-olds (*p* = 0.003) and 10-year-olds (*p* = 0.003), and significantly worse in the Reading Span task compared to 9-year-olds (*p* = 0.008) and 10-year-olds (*p* = 0.010). These differences indicate that younger children had particular difficulty with working memory tasks that required greater executive control.

**Figure 1:**
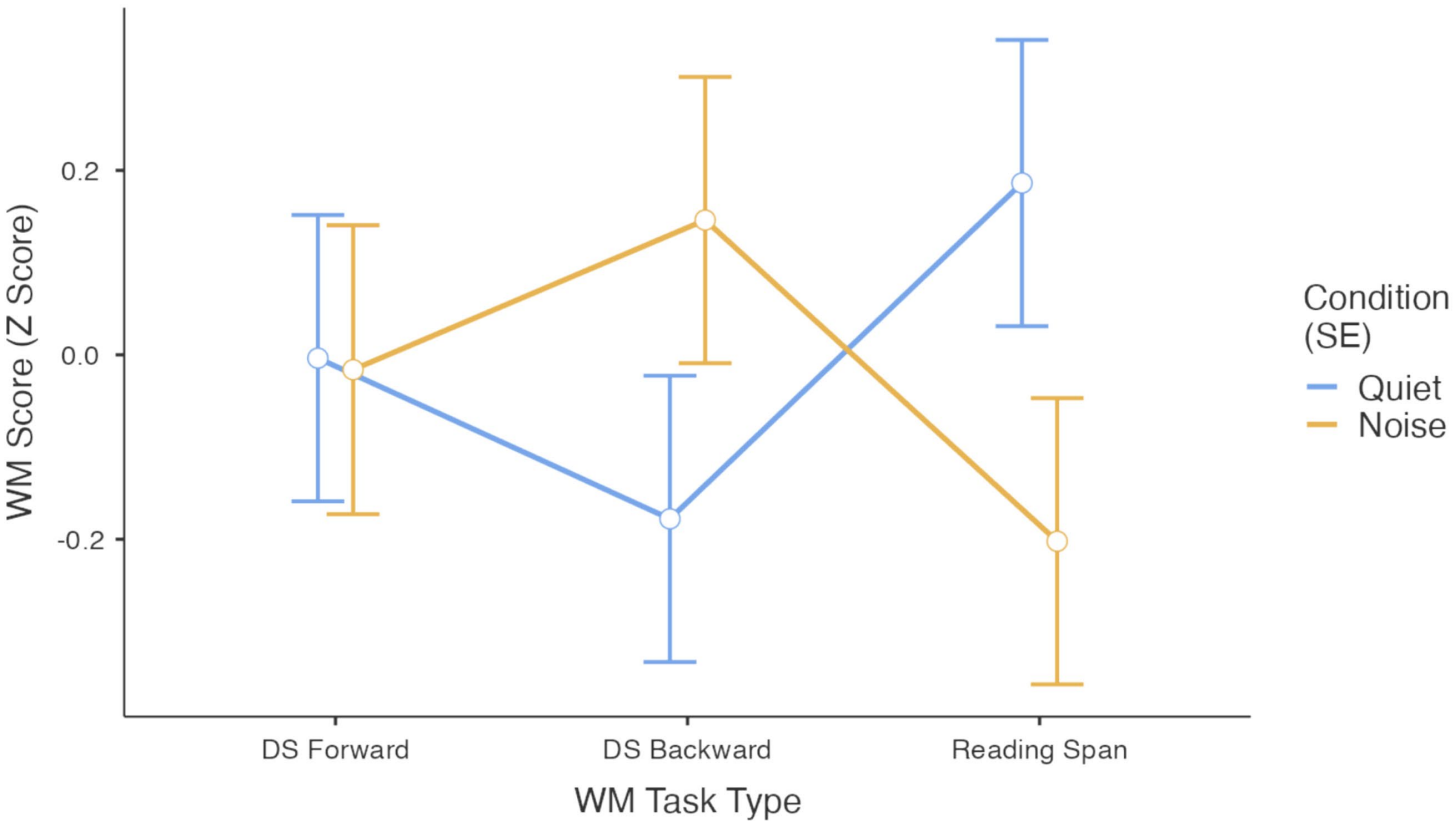
Performance on working memory (WM) tasks (Z scores) across quiet and noise conditions, plotted separately for each WM task type.

Interestingly, the interaction between acoustic condition and WM tasks type was significant, *F*(2, 174) = 3.470, *p* = 0.033 (Figure 1). In line with our *a priori* hypotheses, pairwise comparisons indicated that only in the Reading Span task children’s performance differed significantly between quiet and noise conditions (*p* = 0.046) (Supplementary Table 3S and Figure 1). The fixed effects parameter estimates are reported in Table 2.

**Figure 2:**
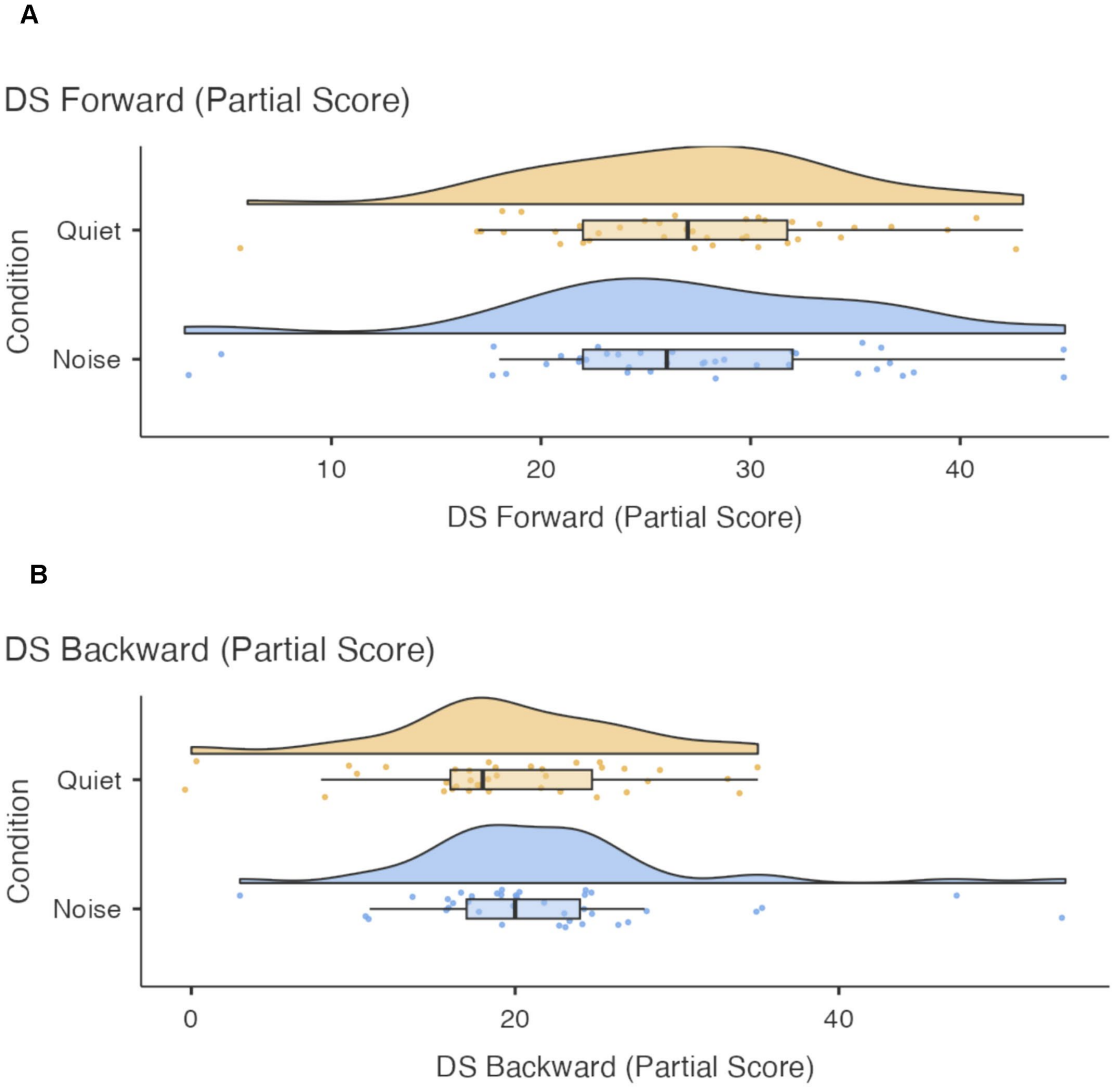
**(A)** Children’s performance on the Digit span forward task. **(B)** Children’s performance on the Digit span backward task.

A visual inspection of Figure 1 suggests different effects of the acoustic condition also on the Digit Span Forward and Digit Span Backward tasks, with performance on the Digit Span Forward task remaining largely stable across conditions whereas performance on the Digit Span Backward appeared to improve in noise than in quiet. However, pairwise comparisons did not reveal significant differences between acoustic conditions for either the Digit Span Forward or Backward tasks (*p* > 0.05). The distribution of children’s scores reported in Figures 2A,B, suggests that a few participants performed exceptionally well on the Backward task in noise, which may explain the pattern observed in Figure 1.

**Figure 3:**
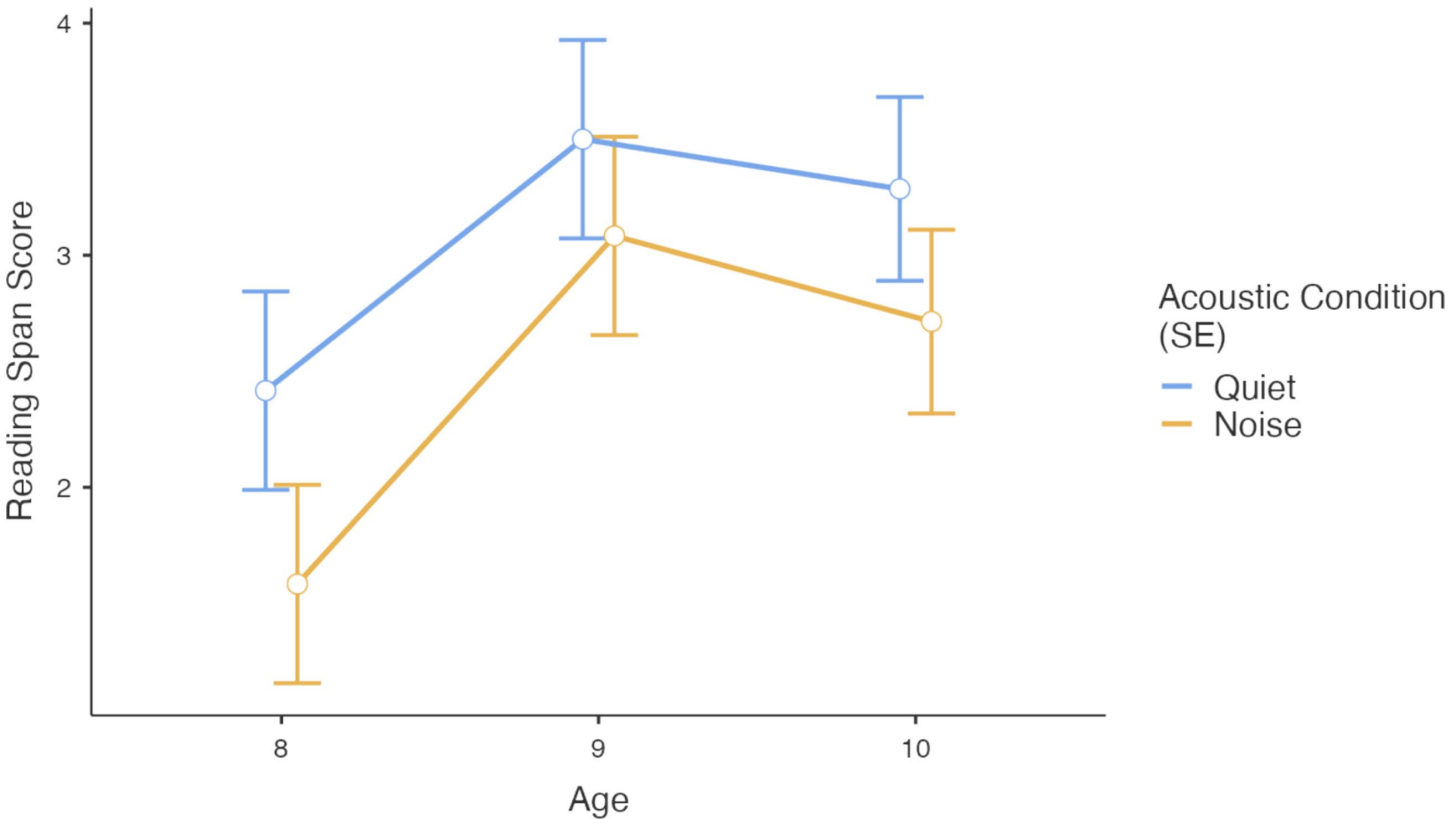
Performance on reading span task (raw score) in quiet and noise conditions by age.

Neither the interaction between age and acoustic condition, nor the three-way interaction between acoustic condition, age group, and working memory task type reached statistical significance, indicating that despite the age-related differences in verbal WM the impact of noise on WM performance was consistent across the three age groups (see Figure 3, Reading Span task).

Table 2 presents the parameter estimates (fixed effects) from the linear mixed-effects model, which complement the omnibus tests reported in the text that assess the overall significance of the main effects and interactions.

Overall, the model explained 34.6% of the variance in verbal working memory performance when including both fixed and random effects (Conditional R^2^ = 0.346), with 14.0% explained by the fixed effects alone (Marginal R^2^ = 0.140). The intra-class correlation coefficient (ICC) indicated that 24% of the variance was attributable to individual differences between participants (ICC = 0.24).

### 3.3 Contribution of visual attention skills on children’s verbal WM performance

As significant condition effects were observed only in the Reading Span task, subsequent analyses focused only on this task. To assess whether children’s sustained visual attention resources contributed to explain their performance on the reading span task in noise, two attentional measures were considered: mean *d’* scores (sensitivity) and mean **C** scores (response bias), both computed by averaging children’s performance on the Visual Search (Faces) and Cancellation (Animals) tasks.

Two separate linear mixed-effects models were conducted. In model 1, mean *d’* scores were included as a covariate, with acoustic condition and age as fixed effects. In model 2, mean C scores were included as a covariate, with acoustic condition and age as fixed effects. Both models also examined the interaction between age group and condition, as well as the interaction between condition and the attentional covariate (*d’* or C).

Since d’ and Criterion were moderately correlated within conditions (r in quiet = −0.631, *p* < 0.001; r in noise = −0.500, *p* = 0.001), variance inflation factors (VIFs) were calculated to assess potential collinearity concerns, that is, whether the correlation between the predictors could make it difficult to estimate their unique contributions to the outcome variable. VIF values were below 2 for both predictors (VIFs = 1.662), indicating acceptable levels of collinearity (Dormann et al., 2013). This supported the decision to include both predictors in the models, as they capture distinct components of attentional control processes.

#### 3.3.1 Model 1: reading span task with *d’* mean as covariate

A linear mixed-effects model was conducted to examine the effects of acoustic condition and age on Reading Span performance, while controlling for individual differences in visual attention sensitivity (mean *d’* scores), which were included as a covariate.

**Figure 4:**
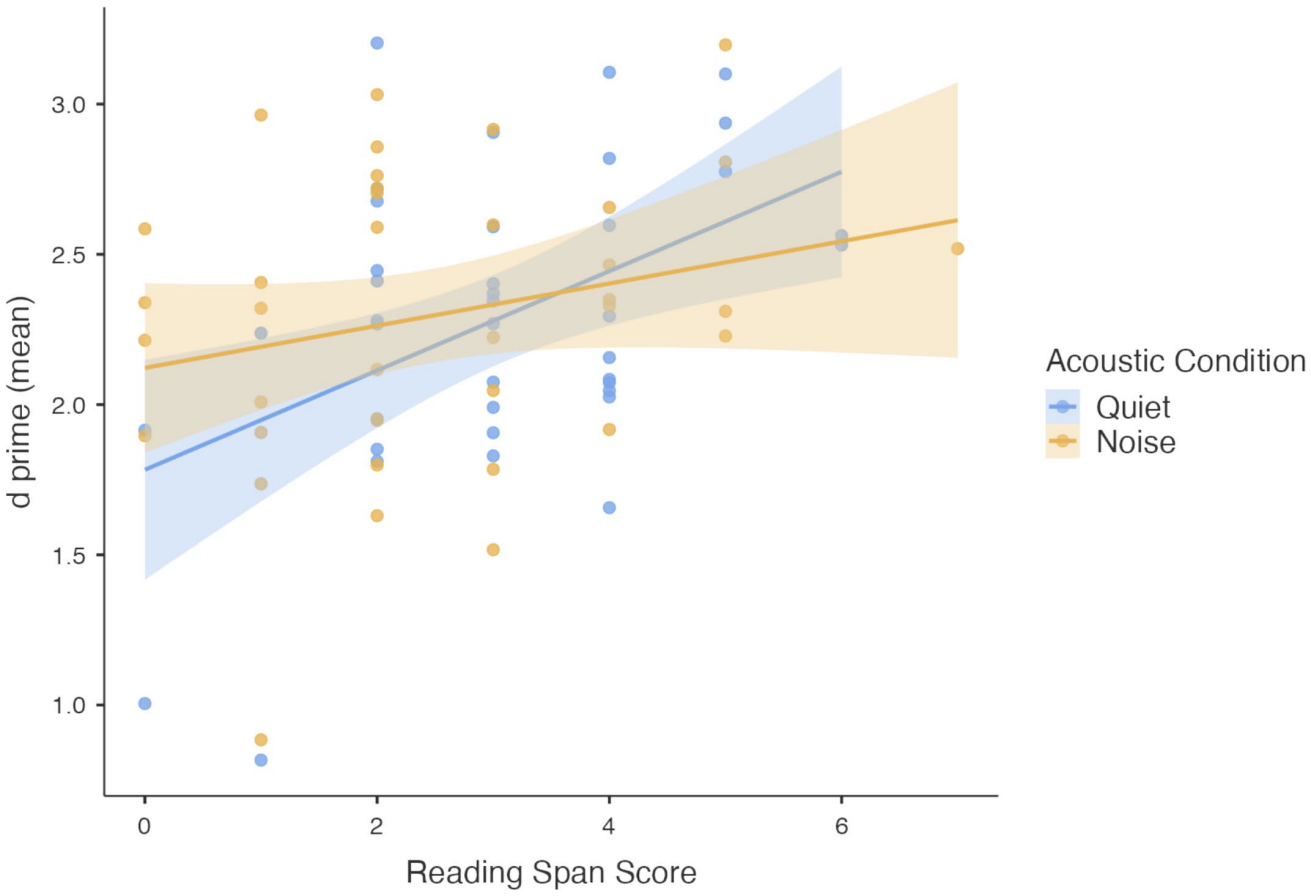
Contribution of attention *d’* mean scores to reading span scores in quiet and noise.

The linear mixed-effects model confirmed a significant main effect of the acoustic condition, *F*(1,33) = 5.200, *p* = 0.029, with a better performance in quiet. Additionally, *d’* mean scores significantly predicted RS performance, *F*(1,66) = 4.467, *p* = 0.038, suggesting that better visual attention abilities are associated with higher working memory skills. Although this was evident especially in quiet (see Figure 4), no significant interactions were found between acoustic condition and *d’* mean scores.

The main effect of age group was not significant, and no significant interactions were found between age and acoustic condition. The parameter estimates (fixed effects) from the linear mixed-effects model are presented in Table 3.

#### 3.3.2 Model 2: reading span task with criterion mean as covariate

A second linear mixed-effects model was conducted to examine the effects of acoustic condition and age on Reading Span performance while controlling for individual differences in response bias (mean C scores), which were included as a covariate.

**Figure 5:**
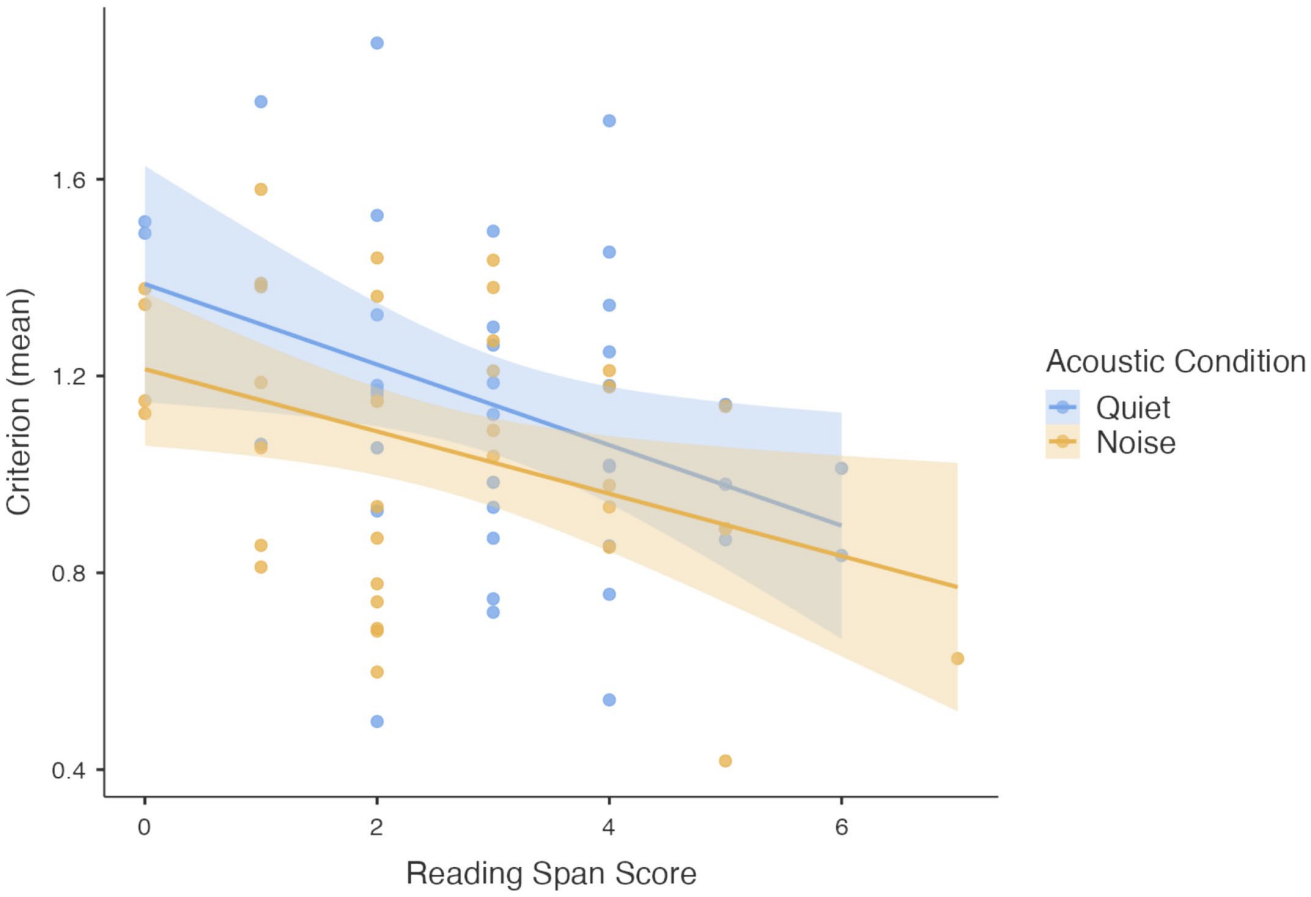
Contribution of attention criterion mean scores to reading span scores in quiet and noise.

The main effect of acoustic condition remained significant, *F*(1,35) = 7.832, *p* = 0.008, confirming again that noise negatively affected RS performance. The Criterion mean scores also had a significant main effect, *F*(1,67) = 8.237, *p* = 0.005, indicating that the decision bias in the visual attention task contributed to RS performance (see also Figure 5). Similar to the previous analyses, the main effect of age was not significant, and no significant interactions were found between age-group and acoustic condition or between acoustic condition and Criterion mean scores. The parameter estimates (fixed effects) from the linear mixed-effects model are presented in Table 4.

To ensure that the effect observed was related to domain - general attentional control mechanisms, we also controlled for the role played by children’s verbal rehearsal skills by including forward digit scores as a covariate. These analyses revealed no significant effect of forward digit scores (i.e., verbal rehearsal abilities) when included as the sole covariate or when added as an additional covariate alongside *d’* or **C**. Importantly, the effects of *d’* and **C** remained significant even when forward digit scores were included as an additional covariate.

## 4 Discussion

The study compared the cognitive interference generated by unintelligible babble noise across verbal working memory tasks with varying cognitive demands and complexity. Although there is evidence suggesting that the Irrelevant Speech Effect (ISE) can occur regardless of whether the speech-noise is intelligible or not (Jones and Macken, 1993; Colle and Welsh, 1976), the specific interference mechanisms of unintelligible speech on verbal working memory remain poorly understood (Gheller et al., 2023). Unlike intelligible speech noise, whose semantic and phonological content can generate automatic linguistic interference on verbal tasks (Dockrell and Shield, 2006; Klatte et al., 2010), unintelligible speech noise, lacking these characteristics, does not automatically occupy listeners in speech encoding processes. However, its speech-like structure could still engage attention and auditory processing pathways, thereby competing with the processing resources required for the verbal rehearsal task at hand (Jones and Macken, 1993; Hughes and Jones, 2001). The present study explored the locus and nature of the potential interference mechanism of unintelligible speech on verbal working memory. The hypothesis of this study was that unintelligible speech would detract domain-general attentional resources from the task, affecting the executive control component of verbal working memory and attentional control mechanisms. The findings partially support this hypothesis.

Although multi-talker babbling resembles speech traces, it did not appear to interfere with the verbal rehearsal mechanisms involved in the Forward Digit Span subtest or with a simple verbal working memory task, such as the Backward Digit Span subtest. The only significant effect of unintelligible multi-talker babbling was observed in the most demanding verbal working memory task, the Reading Span task. These results appear to contrast with findings suggesting that the phonological loop automatically processes both auditory speech and non-speech information external to the verbal memory task during memory performance (Jones and Macken, 1993). However, the characteristics of the unintelligible speech-like audio track used in this study could account for this inconsistency. In the experiment of Jones and Macken’s (1993), a stream of changing tones was used as a distractor during serial recall of visually presented material. In contrast, the multi-talker babbling used in this study consisted of a continuous and steady stream with spectral characteristics similar to white noise. This feature could account for the different locus of interference observed in our study, which was not at the level of the phonological loop but most likely at the level of attentional control mechanisms, as we will discuss later. As a speech-like stimulus, the auditory trace was socially relevant to our participants, potentially making it more challenging for them to sustain attention during the most demanding verbal working memory task, the Reading Span task. In contrast, the speech audio track lacked the dynamic, changing characteristics necessary to disrupt the rehearsal processes of the phonological loop, and thus its effects on the Forward Digit Span task were negligible.

Performance on Reading Span tasks is associated with students’ reading and writing achievements (Arfé et al., 2015; Daneman and Carpenter, 1983). The results of this study thus indicate that irrelevant unintelligible speech, although not significantly disruptive to phonological memory, may still interfere with critical cognitive skills essential for reading-related academic performance. These findings also suggest that the effect of unintelligible speech noise is likely partially mediated by (domain-general) attention mechanisms.

In our analyses we considered whether children’s sustained visual attention abilities could contribute to explain the effects of multi-talker babble noise on verbal working memory tasks. Sensitivity (*d’*) and decision bias (Criterion) averaged across both Visual Search (Faces) and Cancellation (Animals) were included as covariates in the linear mixed-effects models to investigate their contribution to children’s verbal working memory performance. The results revealed that both sensitivity (*d’*) scores and decision bias (Criterion) scores were significantly associated with Reading Span performance. Specifically, higher visual attention sensitivity (*d’* mean scores) was associated with better verbal working memory. Similarly, Criterion scores significantly contributed to reading span performance, indicating that children’s decision-making strategies in visual attention tasks—how conservative or liberal they are in identifying targets— are related to their ability to manage verbal working memory demands. These findings imply that domain-general attentional resources may play a role in children’s capacity to cope with complex verbal working memory tasks. As anticipated, the use of visual attention, rather than auditory attention tasks, supports the conclusion that the attentional effect was domain-general in nature and not specifically tied to auditory attention.

The role played by attentional control mechanisms is suggested by another finding of this study: the interference effect was significant only when verbal task demands were high, as in the Reading Span task. In contrast, children exerted sufficient cognitive control when task demands were lower, such as in Digit Span tasks. Control analyses from this study also confirm that the effect is independent of children’s rehearsal skill, that is, from phonological interference: verbal rehearsal abilities, assessed through children’s performance on the forward digit span task, did not contribute to explain variance in performance on the Reading span task. However, domain-general attentional skills alone cannot explain the disruptive effect of unintelligible speech noise on children’s verbal working memory. While individual differences in sensitivity and Criterion significantly contributed to the overall verbal working memory performance, they did not account for the specific noise effects observed in this study. Therefore, other cognitive factors could explain the interference effects of unintelligible speech. An avenue for future studies is to examine the role of domain-specific attention processes, such as auditory attention. For example, recent findings (Leist et al., 2025) suggest that auditory distraction from unintelligible speech may divert auditory-specific attentional resources away from working memory tasks. However, it remains unclear why this distraction seems less impactful on children’s verbal rehearsal processes, given that auditory interference caused by non-verbal environmental sounds has been found to significantly impair serial recall (Leist et al., 2025).

Recent reviews (Gheller et al., 2023; Mealings, 2022) highlight the inconsistent results across studies examining how verbal noise exposure affects attention. For instance, McGarrigle et al. (2019) found no significant effect of multi-talker babble noise on children’s visual reaction times, whereas Dockrell and Shield (2006) and Fernandes et al. (2019) reported a detrimental effect of multi-talker babble noise on visual attention performance. The attentional consequences of speech noise and their mediation role on verbal working memory should be thus further explored by experimental studies.

As expected, the younger children in this study performed worse than the older children on the verbal working memory tasks. However, the disruptive effects of noise were consistent across all three age groups, which suggests that under stressful conditions noise may be harmful also for the older and cognitively more mature learners. This seems supported by children’s subjective reported perception on cognitive effort self-reports. They indicated that, although children were able to compensate well for noise interference in some tasks, such as the Digit Span Forward and Backward, also in these tasks they found the noise condition more disturbing and tasks under noise more effortful.

This also suggests that increased perceived effort does not necessarily imply decreased performance, which is consistent with the findings of Massonnié et al. (2022) and Hygge (2003), who differentiate between “noise interference” and “noise annoyance.” Noise interference refers to cognitive burden imposed by noise, which can hinder the achievement of task goals by taxing cognitive resources. In contrast, noise annoyance relates to the individual’s capacity to cope with this interference, affecting their emotional reaction to the noise. Thus, while interference addresses the direct impact of noise on task performance, annoyance is related to the strategies employed to mitigate the effects of noise and how much attention is paid to noise itself. Our findings are in line with Massonnié et al. (2022) and Hygge (2003), who emphasized that these two constructs, although related, are distinct. This distinction is reflected in our study, where children were able to maintain task performance despite reporting increased cognitive effort and disturbance, at least in verbal tasks that are less demanding (e.g., digit span task).

This discrepancy between actual performance in noisy conditions and children’s self-reported levels of effort highlights the importance of employing multiple measures when conducting studies on noise effects. For instance, integrating task performance with psychophysiological indexes such as eye pupil dilation (Arfé et al., 2024; Gómez-Merino et al., 2020) could allow for a more exhaustive assessment of underlying cognitive processes that might be taxed when task performance is unaffected.

### 4.1 Limitations and future research

This study has contributed to explaining how unintelligible multi-talker babble noise affects children’s verbal working memory and the role played by attentional skills. However, limitations should be acknowledged and need to be addressed in future research. Firstly, the sample size was relatively small, and all participants were recruited from the same school. Future studies should include a larger and more diverse cohort, including children with developmental or learning disorders, to explore interindividual variability in the effects observed. Secondly, while this study utilized a novel tablet application to assess cognitive functions in an ecological, yet controlled, setting, the digital format might introduce variables that do not exist in traditional paper-pencil tasks, such as interface usability and children’s familiarity with digital devices, which could affect their performance independently of the noise condition. Although we assessed children’s familiarity with electronic devices beforehand, participants were exposed to the application for the first time during the experimental study, without the opportunity for prior training or familiarizing with the tool.

Finally, all tasks remained the same across the two acoustic conditions. While the sessions were one week apart, this interval may not have been sufficient to completely eliminate a learning effect, especially among older children. We controlled this effect by counterbalancing condition order (quiet or noise) across participants, but task items remained the same in both assessments. Future research could address this methodological issue by developing parallel forms of the experimental tasks with different task items (i.e., using different sequences of digits and words).

### 4.2 Conclusion

Multi-talker babble noise is a common feature of children’s everyday environments. However, its effects on cognitive performance and learning have been studied less extensively compared to those of speech noise. This study attempted to address this research gap by examining the effects of unintelligible babble noise on children’s verbal working memory and the role of sustained visual attention skills. The results showed that children experience greater cognitive fatigue under unintelligible noise, which however impacts significantly their performance only in complex cognitive tasks. Although the cognitive effects of unintelligible speech appear related to the diversion of attentional control mechanisms, the specific interference mechanisms remain unclear. The findings, which add to other recent evidence (Leist et al., 2025), suggest that children’s domain-general attentional resources contribute to their verbal working memory performance, but these alone are insufficient to fully explain the interference effect.

By examining children across a critical developmental span for schooling—from 8 to 10 years—our study also deepens our understanding of how the maturation of verbal working memory and attentional resources interacts with auditory disturbances. This age range is pivotal, capturing a phase where cognitive capacities are rapidly growing and differentially susceptible to external stimuli.

Exploring individual differences in susceptibility to noise interference is an important focus of this research area. It not only enhances our understanding of how children cope with noise in learning environments such as classrooms, but also informs educational practices and classroom design.

## Data Availability

The raw data supporting the conclusions of this article will be made available by the authors, without undue reservation.

## References

[ref1] Acheson D. J. MacDonald M. C. (2009). Verbal working memory and language production: common approaches to the serial ordering of verbal information. Psychol. Bull. 135, 50–68. doi: 10.1037/a0014411, PMID: 19210053 PMC3000524

[ref2] Alloway T. P. Gathercole S. E. Willis C. Adams A.-M. (2004). A structural analysis of working memory and related cognitive skills in young children. J. Exp. Child Psychol. 87, 85–106. doi: 10.1016/j.jecp.2003.10.002, PMID: 14757066

[ref3] Arfé B. (2015). Oral and written discourse skills in deaf and hard of hearing children: the role of reading and verbal working memory. Top. Lang. Disord. 35, 180–197. doi: 10.1097/TLD.0000000000000054

[ref4] Arfé B. Fastelli A. (2020). “The influence of explicit and implicit memory processes on the spoken–written language learning of children with cochlear implants” in The Oxford handbook of deaf studies in learning and cognition. eds. Marschark M. Knoors H. (New York, NY: Oxford University Press), 320–335.

[ref5] Arfé B. Rossi C. Sicoli S. (2015). The contribution of verbal working memory to deaf children’s oral and written production. J. Deaf. Stud. Deaf. Educ. 20, 203–214. doi: 10.1093/deafed/env005, PMID: 25802319 PMC4450155

[ref6] Arfé B. Spicciarelli G. Gheller F. Montuori C. Ronconi L. (2022). “Individual differences in children’s cognitive performance in noise.” In Proceedings of the 24th International Congress on Acoustics. pp. 222–229, Gyeongju, 24-28 October 2022.

[ref7] Arfé B. Spicciarelli G. Gheller F. Gómez-Merino N. Martini A. Trevisi P. (2024). Assessing the cognitive effects of noise in children with cochlear implants: a proof of concept study. Rivista di psicolinguistica applicata 24, 63–76. doi: 10.19272/202407702005, PMID: 24183105

[ref8] Baddeley A. (2003). Working memory and language: an overview. J. Commun. Disord. 36, 189–208. doi: 10.1016/S0021-9924(03)00019-4, PMID: 12742667

[ref9] Basner M. Babisch W. Davis A. Brink M. Clark C. Janssen S.. (2014). Auditory and non-auditory effects of noise on health. Lancet 383, 1325–1332. doi: 10.1016/S0140-6736(13)61613-X, PMID: 24183105 PMC3988259

[ref10] Bell R. Mieth L. Röer J. P. Buchner A. (2024). The reverse Mozart effect: music disrupts verbal working memory irrespective of whether you like it or not. J. Cogn. Psychol. 36, 8–27. doi: 10.1080/20445911.2023.2216919

[ref11] Berglund B. Lindvall T. Schwela D. H. World Health Organization (1999). Guidelines for community noise: World Health Organization. Available at: https://iris.who.int/handle/10665/66217 (Accessed July, 2024).

[ref12] Bess F. H. Gustafson S. J. Hornsby B. W. (2014). How hard can it be to listen? Fatigue in school-age children with hearing loss. J. Educ. Audiol. 20, 1–14.

[ref13] Boman E. Enmarker I. (2004). Factors affecting pupils’ noise annoyance in schools: the building and testing of models. Environ. Behav. 36, 207–228. doi: 10.1177/0013916503256644

[ref14] Brehm L. Alday P. M. (2022). Contrast coding choices in a decade of mixed models. J. Mem. Lang. 125:104334. doi: 10.1016/j.jml.2022.104334

[ref15] Brysbaert M. Debeer D. (2025). How to run linear mixed effects analysis for pairwise comparisons? A tutorial and a proposal for the calculation of standardized effect sizes. J. Cogn. 8:5. doi: 10.5334/joc.409, PMID: 39803174 PMC11720698

[ref16] Colle H. A. Welsh A. (1976). Acoustic masking in primary memory. J. Verbal Learn. Verbal Behav. 15, 17–31. doi: 10.1016/S0022-5371(76)90003-7

[ref17] Connolly D. Dockrell J. Shield B. Conetta R. Mydlarz C. Cox T. (2019). The effects of classroom noise on the reading comprehension of adolescents. J. Acoust. Soc. Am. 145, 372–381. doi: 10.1121/1.5087126, PMID: 30710912

[ref18] Conway A. R. Kane M. J. Bunting M. F. Hambrick D. Z. Wilhelm O. Engle R. W. (2005). Working memory span tasks: a methodological review and user’s guide. Psychon. Bull. Rev. 12, 769–786. doi: 10.3758/BF03196772, PMID: 16523997

[ref19] Cutugno F. Prosser S. Turrini M. (2000). Audiometria vocale – vol. I. ed. GN. ReSound Italia 2000.

[ref20] Daneman M. Carpenter P. A. (1980). Individual differences in working memory and reading. J. Verbal Learn. Verbal Behav. 19, 450–466. doi: 10.1016/S0022-5371(80)90312-6

[ref21] Daneman M. Carpenter P. A. (1983). Individual differences in integrating information between and within sentences. J. Exp. Psychol. Learn. Mem. Cogn. 9, 561–584. doi: 10.1037/0278-7393.9.4.561, PMID: 40165438

[ref22] Dockrell J. E. Shield B. M. (2006). Acoustical barriers in classrooms: the impact of noise on performance in the classroom. Br. Educ. Res. J. 32, 509–525. doi: 10.1080/01411920600635494

[ref23] Donolato E. Giofrè D. Mammarella I. C. (2017). Differences in verbal and visuospatial forward and backward order recall: a review of the literature. Front. Psychol. 8:663. doi: 10.3389/fpsyg.2017.00663, PMID: 28522982 PMC5415597

[ref24] Dormann C. F. Elith J. Bacher S. Buchmann C. Carl G. Carré G.. (2013). Collinearity: a review of methods to deal with it and a simulation study evaluating their performance. Ecography 36, 27–46. doi: 10.1111/j.1600-0587.2012.07348.x

[ref25] Elliott E. M. (2002). The irrelevant-speech effect and children: theoretical implications of developmental change. Mem. Cogn. 30, 478–487. doi: 10.3758/BF03194948, PMID: 12061768

[ref26] Elliott E. M. Hughes R. W. Briganti A. Joseph T. N. Marsh J. E. Macken B. (2016). Distraction in verbal short-term memory: insights from developmental differences. J. Mem. Lang. 88, 39–50. doi: 10.1016/j.jml.2015.12.008

[ref27] Fernandes R. A. Vidor D. C. G. M. Oliveira A. A. D. (2019). The effect of noise on attention and performance in reading and writing tasks. CoDAS 31:e20170241. doi: 10.1590/2317-1782/20182017241, PMID: 31483038

[ref28] Gallucci M. (2019). GAMLj: general analyses for linear models. [jamovi module]. Available online at: https://gamlj.github.io/ (Accessed January, 2025).

[ref29] Gathercole S. E. Pickering S. J. Ambridge B. Wearing H. (2004). The structure of working memory from 4 to 15 years of age. Dev. Psychol. 40, 177–190. doi: 10.1037/0012-1649.40.2.177, PMID: 14979759

[ref30] Gheller F. Spicciarelli G. Battagliarin L. Cappelletti F. Di Bella A. Romagnoni P.. (2024). Effects of noise on the cognitive performance of primary school children. Rivista Italiana di Acustica 48, 81–90. doi: 10.3280/ria1-2024oa17501

[ref31] Gheller F. Spicciarelli G. Scimemi P. Arfé B. (2023). The effects of noise on children’s cognitive performance: a systematic review. Environ. Behav. 55, 698–734. doi: 10.1177/00139165241245823

[ref32] Giofrè D. Stoppa E. Ferioli P. Pezzuti L. Cornoldi C. (2016). Forward and backward digit span difficulties in children with specific learning disorder. J. Clin. Exp. Neuropsychol. 38, 478–486. doi: 10.1080/13803395.2015.1125454, PMID: 26727043

[ref33] Gómez-Merino N. Gheller F. Spicciarelli G. Trevisi P. (2020). Pupillometry as a measure for listening effort in children: a review. Hear. Balance Commun. 18, 152–158. doi: 10.1080/21695717.2020.1807256

[ref34] Granziol U. Rabe M. Gallucci M. Spoto A. Vidotto G. (2025). Not another post hoc paper: a new look at contrast analysis and planned comparisons. Adv. Methods Pract. Psychol. Sci. 8:3110. doi: 10.1177/25152459241293110, PMID: 40270978

[ref35] Grebennikov L. Wiggins M. (2006). Psychological effects of classroom noise on early childhood teachers. Aust. Educ. Res. 33, 35–53. doi: 10.1007/BF03216841

[ref36] Guerra G. Tijms J. Vaessen A. Tierney A. Dick F. Bonte M. (2021). Loudness and intelligibility of irrelevant background speech differentially hinder children's short story reading. Mind Brain Educ. 15, 77–87. doi: 10.1111/mbe.12264

[ref37] Howard C. S. Munro K. J. Plack C. J. (2010). Listening effort at signal-to-noise ratios that are typical of the school classroom. Int. J. Audiol. 49, 928–932. doi: 10.3109/14992027.2010.520036, PMID: 21047295

[ref38] Hughes R. Jones D. M. (2001). The intrusiveness of sound: laboratory findings and their implications for noise abatement. Noise Health 4, 51–70, PMID: 12678935

[ref39] Hygge S. (2003). Classroom experiments on the effects of different noise sources and sound levels on long-term recall and recognition in children. Appl. Cogn. Psychol. 17, 895–914. doi: 10.1002/acp.926

[ref40] Jianxin P. Peng J. (2018). The effects of the noise and reverberation on the working memory span of children. Arch. Acoust. 43, 123–128. doi: 10.24425/118087

[ref41] Jones D. M. Macken W. J. (1993). Irrelevant tones produce an irrelevant speech effect: implications for phonological coding in working memory. J. Exp. Psychol. Learn. Mem. Cogn. 19, 369–381. doi: 10.1037/0278-7393.19.2.369, PMID: 40165438

[ref42] Jones D. M. Miles C. Page J. (1990). Disruption of proofreading by irrelevant speech: effects of attention, arousal or memory? Appl. Cogn. Psychol. 4, 89–108. doi: 10.1002/acp.2350040203

[ref43] Kam J. (2017). The wandering mind: how the brain allows us to mentally wander off to another time and place. Front. Young Minds 5:25. doi: 10.3389/frym.2017.00025

[ref44] Klatte M. Bergström K. Lachmann T. (2013). Does noise affect learning? A short review on noise effects on cognitive performance in children. Front. Psychol. 4:55965. doi: 10.3389/fpsyg.2013.00578, PMID: 24009598 PMC3757288

[ref45] Klatte M. Lachmann T. Schlittmeier S. Hellbrück J. (2010). The irrelevant sound effect in short-term memory: is there developmental change? Eur. J. Cogn. Psychol. 22, 1168–1191. doi: 10.1080/09541440903378250

[ref46] Korkman M. Kirk U. Kemp S. (2011) in NEPSY-ii – Second edition. Italian adaptation. eds. Urgesi C. Fabbro F. (Firenze, Italy: Giunti O.S).

[ref47] Kristiansen J. Lund S. P. Nielsen P. M. Persson R. Shibuya H. (2011). Determinants of noise annoyance in teachers from schools with different classroom reverberation times. J. Environ. Psychol. 31, 383–392. doi: 10.1016/j.jenvp.2011.08.005

[ref48] Leist L. Lachmann T. Klatte M. (2025). Impact of irrelevant speech and non-speech sounds on serial recall of verbal and spatial items in children and adults. Sci. Rep. 15:1951. doi: 10.1038/s41598-025-85855-w, PMID: 39809959 PMC11732993

[ref49] Lundquist P. Holmberg K. Landstrom U. (2000). Annoyance and effects on work from environmental noise at school. Noise Health 2, 39–46, PMID: 12689460

[ref50] Mama Y. Fostick L. Icht M. (2018). The impact of different background noises on the production effect. Acta Psychol. 185, 235–242. doi: 10.1016/j.actpsy.2018.03.002, PMID: 29559082

[ref51] Martin R. C. Wogalter M. S. Forlano J. G. (1988). Reading comprehension in the presence of unattended speech and music. J. Mem. Lang. 27, 382–398. doi: 10.1016/0749-596X(88)90063-0

[ref52] Massonnié J. Frasseto P. Mareschal D. Kirkham N. Z. (2022). Learning in noisy classrooms: Children’s reports of annoyance and distraction from noise are associated with individual differences in mind-wandering and switching skills. Environ. Behav. 54, 58–88. doi: 10.1177/0013916520950277

[ref53] McGarrigle R. Gustafson S. J. Hornsby B. W. Bess F. H. (2019). Behavioral measures of listening effort in school-age children: examining the effects of signal-to-noise ratio, hearing loss, and amplification. Ear Hear. 40, 381–392. doi: 10.1097/AUD.0000000000000623, PMID: 29905670

[ref54] Mealings K. (2022). Classroom acoustics and cognition: a review of the effects of noise and reverberation on primary school children’s attention and memory. Building Acoust. 29, 401–431. doi: 10.1177/1351010X221104892

[ref55] Nakagawa S. Schielzeth H. (2013). A general and simple method for obtaining R2 from generalized linear mixed-effects models. Methods Ecol. Evol. 4, 133–142. doi: 10.1111/j.2041-210x.2012.00261.x

[ref56] Olsthoorn N. M. Andringa S. Hulstijn J. H. (2014). Visual and auditory digit-span performance in native and non-native speakers. Int. J. Biling. 18, 663–673. doi: 10.1177/1367006912466314

[ref57] Perham N. Sykora M. (2012). Disliked music can be better for performance than liked music. Appl. Cogn. Psychol. 26, 550–555. doi: 10.1002/acp.2826, PMID: 40275783

[ref58] Perham N. Vizard J. (2011). Can preference for background music mediate the irrelevant sound effect? Appl. Cogn. Psychol. 25, 625–631. doi: 10.1002/acp.1731

[ref59] Salamé P. Baddeley A. (1989). Effects of background music on phonological short-term memory. Q. J. Exp. Psychol. 41, 107–122. doi: 10.1080/14640748908402355

[ref60] Scerif G. Longhi E. Cole V. Karmiloff-Smith A. Cornish K. (2012). Attention across modalities as a longitudinal predictor of early outcomes: the case of fragile X syndrome. J. Child Psychol. Psychiatry 53, 641–650. doi: 10.1111/j.1469-7610.2011.02515.x, PMID: 22211574

[ref61] Schweppe J. Knigge J. (2020). Irrelevant music: how suprasegmental changes of a melody’s tempo and mode affect the disruptive potential of music on serial recall. Mem. Cogn. 48, 982–993. doi: 10.3758/s13421-020-01037-1, PMID: 32385674 PMC7381464

[ref62] Shield B. M. Dockrell J. E. (2004). External and internal noise surveys of London primary schools. J. Acoust. Soc. Am. 115, 730–738. doi: 10.1121/1.1635837, PMID: 15000185

[ref63] Shield B. M. Dockrell J. E. (2008). The effects of environmental and classroom noise on the academic attainments of primary school children. J. Acoust. Soc. Am. 123, 133–144. doi: 10.1121/1.2812596, PMID: 18177145

[ref64] Sjödin F. Kjellberg A. Knutsson A. Landström U. Lindberg L. (2012). Noise exposure and auditory effects on preschool personnel. Noise Health 14, 72–82. doi: 10.4103/1463-1741.95135, PMID: 22517307

[ref65] Stallen P. J. M. (1999). A theoretical framework for environmental noise annoyance. Noise Health 1, 69–80, PMID: 12689501

[ref66] Stanislaw H. Todorov N. (1999). Calculation of signal detection theory measures. Behav. Res. Methods Instrum. Comput. 31, 137–149. doi: 10.3758/BF03207704, PMID: 10495845

[ref67] The jamovi project. (2024). Jamovi. (version 2.6) [computer software]. Available online at: https://www.jamovi.org (Accessed July, 2024).

[ref68] Tractenberg R. E. Freas C. E. (2007). Benchmarking a computerized test of immediate verbal memory. Behav. Res. Methods 39, 863–869. doi: 10.3758/BF03192980, PMID: 18183902

[ref69] Vasilev M. R. Liversedge S. P. Rowan D. Kirkby J. A. Angele B. (2019). Reading is disrupted by intelligible background speech: evidence from eye-tracking. J. Exp. Psychol. Hum. Percept. Perform. 45, 1484–1512. doi: 10.1037/xhp0000680, PMID: 31436455

[ref70] Vettori G. Di Leonardo L. Secchi S. Astolfi A. Bigozzi L. (2022). Primary school children’s verbal working memory performances in classrooms with different acoustic conditions. Cogn. Dev. 64:101256. doi: 10.1016/j.cogdev.2022.101256

[ref71] Wechsler D. (2003). Wechsler intelligence scale for children-fourth edition (WISC-IV). San Antonio, TX: Psychological Corporation.

[ref72] Weitzner D. S. Calamia M. Hill B. D. Elliott E. M. (2022). Examining an alternative scoring procedure for a clinical working memory measure. Assessment 29, 1756–1764. doi: 10.1177/10731911211032270, PMID: 34282641

[ref73] Wells E. L. Kofler M. J. Soto E. F. Schaefer H. S. Sarver D. E. (2018). Assessing working memory in children with ADHD: minor administration and scoring changes may improve digit span backward’s construct validity. Res. Dev. Disabil. 72, 166–178. doi: 10.1016/j.ridd.2017.10.024, PMID: 29156389 PMC5743590

[ref74] Welsh M. C. Pennington B. F. Groisser D. B. (1991). A normative-developmental study of executive function: a window on prefrontal function in children. Dev. Neuropsychol. 7, 131–149. doi: 10.1080/87565649109540483

[ref75] Zhu J. Chen H. (2013). Clinical utility of cancellation on the WISC-IV. J. Psychoeduc. Assess. 31, 527–537. doi: 10.1177/0734282913480865

[ref76] Zoccolotti P. De Luca M. Di Filippo G. Judica A. Martelli M. (2009). Reading development in an orthographically regular language: effects of length, frequency, lexicality and global processing ability. Read. Writ. 22, 1053–1079. doi: 10.1007/s11145-008-9144-8

