# Supplementary material for: The effect of unintelligible speech noise on children’s verbal working memory performance
Source: Front Psychol. 2025 May 26;16:1565112. doi: 10.3389/fpsyg.2025.1565112 (PMC12146341; doi:10.3389/fpsyg.2025.1565112)
Supplement: Supplementary file 1 [file Supplementary_file_1.docx]

Supplementary Material

# Supplementary Tables

Table 1S. Main effect of age: Pairwise comparisons.

| Comparisons | | | Estimate | SE | *t* | *df* | *p* |
| --- | --- | --- | --- | --- | --- | --- | --- |
| 8 years | vs | 9 years | -0.576 | 0.236 | -2.442 | 35 | .020 |
| 8 years | vs | 10 years | -0.644 | 0.228 | -2.829 | 35 | .008 |
| 9 years | vs | 10 years | -0.068 | 0.228 | -.298 | 35 | .768 |

Table 2S. Interaction between age and working memory task type: Pairwise comparisons.

| Comparisons | | | Estimate | SE | *t* | *df* | *p* |
| --- | --- | --- | --- | --- | --- | --- | --- |
| 8 years DS Forward | vs | 8 years DS Backward | 0.499 | 0.24 | 2.076 | 174.117 | .039 |
| 8 years DS Forward | vs | 8 years Reading Span | 0.367 | 0.24 | 1.53 | 174.117 | .128 |
| 8 years DS Forward | vs | 9 years DS Forward | 0.02 | 0.307 | 0.067 | 91.203 | .947 |
| 8 years DS Forward | vs | 9 years DS Backward | -0.424 | 0.307 | -1.382 | 91.203 | .17 |
| 8 years DS Forward | vs | 9 years Reading Span | -0.459 | 0.307 | -1.496 | 91.203 | .138 |
| 8 years DS Forward | vs | 10 years DS Forward | -0.379 | 0.297 | -1.273 | 92.757 | .206 |
| 8 years DS Forward | vs | 10 years DS Backward | -0.415 | 0.296 | -1.403 | 91.203 | .164 |
| 8 years DS Forward | vs | 10 years Reading Span | -0.272 | 0.296 | -0.921 | 91.203 | .359 |
| 8 years DS Backward | vs | 8 years Reading Span | -0.131 | 0.24 | -0.546 | 174.117 | .586 |
| 8 years DS Backward | vs | 9 years DS Forward | -0.478 | 0.307 | -1.558 | 91.203 | .123 |
| 8 years DS Backward | vs | 9 years DS Backward | -0.923 | 0.307 | -3.007 | 91.203 | .003 |
| 8 years DS Backward | vs | 9 years Reading Span | -0.958 | 0.307 | -3.121 | 91.203 | .002 |
| 8 years DS Backward | vs | 10 years DS Forward | -0.877 | 0.297 | -2.949 | 92.757 | .004 |
| 8 years DS Backward | vs | 10 years DS Backward | -0.913 | 0.296 | -3.09 | 91.203 | .003 |
| 8 years DS Backward | vs | 10 years Reading Span | -0.771 | 0.296 | -2.608 | 91.203 | .011 |
| 8 years Reading Span | vs | 9 years DS Forward | -0.347 | 0.307 | -1.131 | 91.203 | .261 |
| 8 years Reading Span | vs | 9 years DS Backward | -0.791 | 0.307 | -2.579 | 91.203 | .011 |
| 8 years Reading Span | vs | 9 years Reading Span | -0.826 | 0.307 | -2.694 | 91.203 | .008 |
| 8 years Reading Span | vs | 10 years DS Forward | -0.746 | 0.297 | -2.508 | 92.757 | .014 |
| 8 years Reading Span | vs | 10 years DS Backward | -0.782 | 0.296 | -2.646 | 91.203 | .01 |
| 8 years Reading Span | vs | 10 years Reading Span | -0.64 | 0.296 | -2.164 | 91.203 | .033 |
| 9 years DS Forward | vs | 9 years DS Backward | -0.444 | 0.24 | -1.851 | 174.117 | .066 |
| 9 years DS Forward | vs | 9 years Reading Span | -0.479 | 0.24 | -1.997 | 174.117 | .047 |
| 9 years DS Forward | vs | 10 years DS Forward | -0.399 | 0.297 | -1.342 | 92.757 | .183 |
| 9 years DS Forward | vs | 10 years DS Backward | -0.435 | 0.296 | -1.473 | 91.203 | .144 |
| 9 years DS Forward | vs | 10 years Reading Span | -0.293 | 0.296 | -0.991 | 91.203 | .324 |
| 9 years DS Backward | vs | 9 years Reading Span | -0.035 | 0.24 | -0.146 | 174.117 | .884 |
| 9 years DS Backward | vs | 10 years DS Forward | 0.045 | 0.297 | 0.152 | 92.757 | .879 |
| 9 years DS Backward | vs | 10 years DS Backward | 0.009 | 0.296 | 0.031 | 91.203 | .976 |
| 9 years DS Backward | vs | 10 years Reading Span | 0.152 | 0.296 | 0.513 | 91.203 | .609 |
| 9 years Reading Span | vs | 10 years DS Forward | 0.08 | 0.297 | 0.27 | 92.757 | .788 |
| 9 years Reading Span | vs | 10 years DS Backward | 0.044 | 0.296 | 0.149 | 91.203 | .882 |
| 9 years Reading Span | vs | 10 years Reading Span | 0.187 | 0.296 | 0.631 | 91.203 | .529 |
| 10 years DS Forward | vs | 10 years DS Backward | -0.036 | 0.225 | -0.161 | 174.591 | .872 |
| 10 years DS Forward | vs | 10 years Reading Span | 0.106 | 0.225 | 0.473 | 174.591 | .637 |
| 10 years DS Backward | vs | 10 years Reading Span | 0.142 | 0.222 | 0.641 | 174.117 | .522 |

**Note.** DS = Digit Span.

Table 3S. Performance in noise and quiet across working memory tasks: Estimated marginal means and pairwise comparisons.

| WM Task Type | Condition | EMM | SE | *df* | 95% CI |
| --- | --- | --- | --- | --- | --- |
| DS Forward | Quiet | 0.00314 | 0.162 | 158 | [-0.316, 0.322] |
|  | Noise | -0.00227 | 0.163 | 161 | [-0.325, 0.32] |
| DS Backward | Quiet | -0.15568 | 0.162 | 158 | [-0.475, 0.163] |
|  | Noise | 0.15568 | 0.162 | 158 | [-0.163, 0.475] |
| Reading Span | Quiet | 0.19364 | 0.162 | 158 | [-0.125, 0.513] |
|  | Noise | -0.19364 | 0.162 | 158 | [-0.513, 0.125] |

# Note. DS = Digit Span; EMM = Estimated Marginal Means; CI = Confidence interval.

| Comparisons | | | Estimate | SE | *t* | *df* | *p* |
| --- | --- | --- | --- | --- | --- | --- | --- |
| DS Forward Quiet | vs | DS Forward Noise | 0.0054 | 0.195 | 0.028 | 184 | .978 |
| DS Backward Quiet | vs | DS Backward Noise | -0.3114 | 0.193 | -1.611 | 184 | .109 |
| Reading Span Quiet | vs | Reading Span Noise | 0.3873 | 0.193 | 2.004 | 184 | .046 |
